# Illumina sequencing‐based analysis of sediment bacteria community in different trophic status freshwater lakes

**DOI:** 10.1002/mbo3.450

**Published:** 2017-02-07

**Authors:** Yu Wan, Xiaohong Ruan, Yaping Zhang, Rongfu Li

**Affiliations:** ^1^ Key Laboratory of Surficial Geochemistry Ministry of Education Nanjing University Nanjing China; ^2^ School of Earth Science and Engineering Nanjing University Nanjing China

**Keywords:** bacteria communities and biodiversity, Illumina Sequencing, Lake Taihu, sediment, trophic status of aquatic ecosystem

## Abstract

Sediment bacterial community is the main driving force for nutrient cycling and energy transfer in aquatic ecosystem. A thorough understanding of the community's spatiotemporal variation is critical for us to understand the mechanisms of cycling and transfer. Here, we investigated the sediment bacterial community structures and their relations with environmental factors, using Lake Taihu as a model system to explore the dependence of biodiversity upon trophic level and seasonality. To combat the limitations of conventional techniques, we employed Illumina MiSeq Sequencing and LeFSe cladogram to obtain a more comprehensive view of the bacterial taxonomy and their variations of spatiotemporal distribution. The results uncovered a 1,000‐fold increase in the total amount of sequences harvested and a reverse relationship between trophic level and the bacterial diversity in most seasons of a year. A total of 65 phyla, 221 classes, 436 orders, 624 families, and 864 genera were identified in the study area. *Delta‐proteobacteria* and *gamma‐proteobacteria* prevailed in spring/summer and winter, respectively, regardless trophic conditions; meanwhile, the two classes dominated in the eutrophication and mesotrophication lake regions, respectively, but exclusively in the Fall. For LEfSe analysis, bacterial taxon that showed the strongest seasonal or spatial variation, majority had the highest abundance in spring/summer or medium eutrophication region, respectively. Pearson's correlation analysis indicated that 5 major phyla and 18 sub‐phylogenetic groups showed significant correlation with trophic status. Canonical correspondence analysis further revealed that porewater NH_4_^+^‐N as well as sediment TOM and NO_x_‐N are likely the dominant environmental factors affecting bacterial community compositions.

## 1 Introduction

Nutrient exchange between water and sediments plays a critical role in adjusting the trophic level of the water body in the aquatic ecosystem (Sinkko et al., 2013). It is well‐documented that such exchange, such as the transformation and biogeochemical cycling of nitrogen and phosphorus, are driven by sediment microorganisms (Hupfer, Gloess, & Grossart, 2007; Hou, Song, Cao, & Zhou, 2013). Because the spatial and temporal distribution of these microbes is controlled by physiochemical conditions of the sediments, temperature, nitrogen level, and organic matter in particular (Haller et al., 2011; Song, Li, Du, Wang, & Ding, 2012), a shift in sediment bacterial communities can provide important insights into environmental changes in the local ecosystem.

Bacterial community is characterized by its structure and biodiversity which have been well studied so far by conventional experimental techniques such as polymerase chain reaction‐denaturing gradient gel electrophoresis (PCR‐DGGE) and clone library techniques. These investigations helped to establish a broad understanding concerning microbial community's temporal and spatial distribution patterns. For example, a PCR‐DGGE/clone library study of the bacterial community in Sitka stream, Czech Republic found that most of the *mcrA* gene clones showed low affiliation with known species and probably represented genes of novel methanogenic archeal genera/species (Rulik et al., 2013). Another research in the Yangtze Delta (Huang, Xie, Yuan, Xu, & Lu, 2014), using the same technique found the number of total cultivable bacteria in an estuary reservoir was significantly lower than that of the main river. Despite the advancement, the knowledge obtained by these studies may have its limitation because the low‐throughput methods employed often underestimate the overall diversity and lack the ability to detect rare species in complicated environmental systems. For example, Berdjeb, Pollet, Chardon, and Jacquet (2013) used the similar methods to examine the archaeal community structure in two neighboring peri‐alpine lakes of different trophic status but found no spatiotemporal dynamics in their study area, suggesting the potential inadequacy of the conventional techniques to probing the complexity of biodiversity and community structure in natural environments.

Compared to the conventional methods, high throughput sequencing has the advantage of being able to generate multi‐million sequences and thousands of Operational Taxonomic Units (OTUs) in environmental samples. For example, Conrad et al. (2014) used pyrosequencing to obtained more than 1000 bacterial OTUs in the sediment of Amazon region and found that rewetting of the sediments resulted in a dramatic increase of the relative abundance of *Clostridiales*. The chosen study area, Lake Taihu (2,338 km^2^), is highly heterogeneous in the trophic levels due to the difference in river input to different regions. As such, the water body in the lake can be divided into different ecological types based upon trophic status and plankton community structure. Spatial variation of bacterial communities in the lake sediments was documented by a number of researchers but no consensus has been reached so far. For example, Liu et al. (2009) reported the absence of *Actinobacteria* in the eutrophied area of the lake, but was contradicted by Chen et al. (2015), where the authors detected as much as 5% abundance for this phylum. Similar inconsistency can be found for the spatial distribution of *Cyanobacteria*,* alpha‐proteobacteria*, and *Planctomycetes* upon comparing the results by Shao et al. (2011) and Chen et al. (2015). On the vertical dimension, Ye et al. (2009) reported similarity of bacterial communities in different layers of sediments taken from Meiliang Bay, but Shao et al. (2013) in a later work discovered the variation of bacterial community and an overall decrease of biodiversity with depth in Meiliang Bay. A literature review indicates such disagreement may have originated largely from the limitations of the clone library because most of these previous studies employed the conventional analytical techniques. In this study, we assessed the sediment bacterial community in a lake with known trophic gradient used a high‐throughput sequencing method (Illumina MiSeq) to circumvent the technical limitations of the traditional methods. For data processing, we used Linear discriminative analysis Effect Size (LEfSe) to recover the spatiotemporal variations of the bacterial community. The aim of this study is to dissect the bacterial community, using the high‐throughput sequencing technique (1) to determine the relations of sediment bacterial taxa with the trophic status of the lake water and sedimentary environmental factors (2) and to provide powerful evidences for further elucidation of the nutrients cycle and accumulation mechanism driven by bacteria in aquatic ecosystem.

## 2 Materials and Methods

### 2.1 Sampling site and procedure

**Figure 1:**
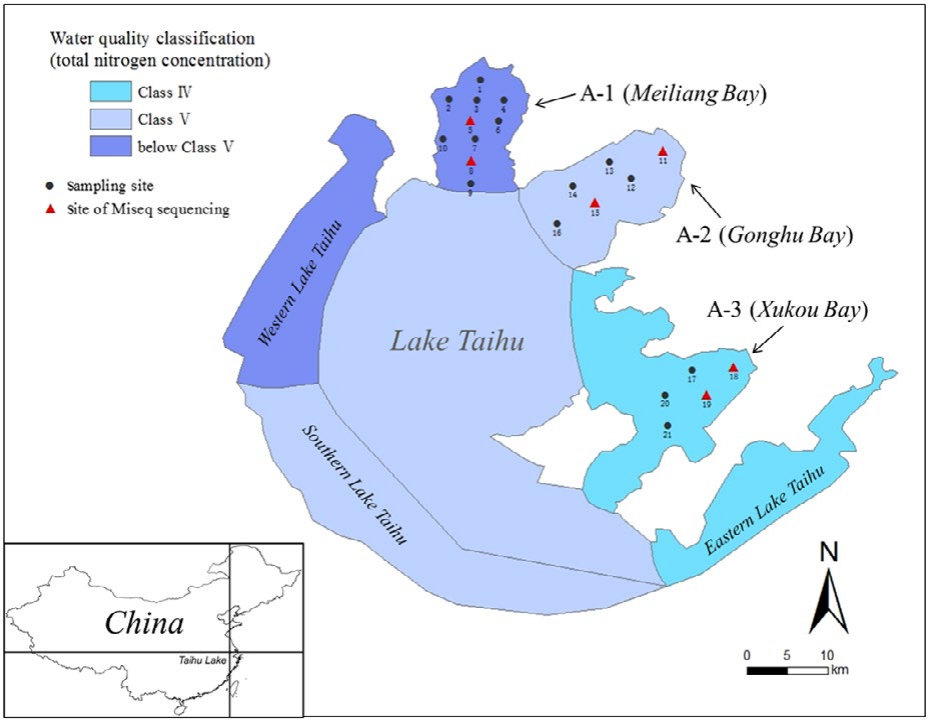
The 21 sampling sites in the different trophic statuses regions of Lake Taihu. Water quality classification of the lake regions referred to the Standard GB3838‐2002 of China. The data of total nitrogen concentration in the figure is from *the Health Status Report of Taihu Lake 2015* in Water Resources Department of Jiangsu Province. Sites 1 to 10, sites 11 to 16, and sites 17 to 21 were located in the Meiliang Bay (A‐1), Gonghu Bay (A‐2), and Xukou Bay (A‐3), respectively. Site 5, 8, 11, 15, 18, and 19 were performed further analysis by Illumina MiSeq sequencing

The study area (Figure 1) is located at the north to east side of the Lake Taihu with total nitrogen decreasing from Meiliang Bay (region A‐1, north), to Gonghu Bay (region A‐2, northeast), and finally to Xukou Bay (region A‐3, east). Area A‐1 is highly enriched in nutrients and has frequent algal blooming incidents. In contrast, the low nutrient waterbody in A‐3 is characterized by submersed vegetation and diverse communities of fishes and invertebrates and, in fact, is a drinking water source for local communities. The water in A‐2 was similar to that in A‐1 till about 15 years ago but has since improved its quality due to the interference of the local government.

Sample collection was carried out in the Fall of 2014, and in Winter, Spring, and Summer of 2015. For sediments, loose sediment samples in the depth of less than 5 cm was collected using a 1/16 m^2^ Petersen grab sampler. Triplicate samples from three separate grabs were homogenized to generate one composite sample in each sampling site. Water samples were taken together at the same locations. All samples were immediately stored in an icebox and transported back to the lab within 3 hr. Once in the lab, an aliquot of the sediment samples was placed in a 15 ml sterile centrifuge tube at −80°C until DNA extraction was carried out. The remaining portion was further processed (freeze‐dried to collect sediment particles, and centrifuged to collect the pore water) for physicochemical analyses.

### 2.2 Physicochemical analyses

Seventeen physicochemical parameters of the overlying water, pore water, and freeze dried sediments were analyzed (Table 1).

**Table 1:** Physicochemical parameters of the overlying water, pore water, and freeze dried sediments

	Physicochemical Parameters
Sediment	Total nitrogen (S_TN), ammonium nitrogen (S_NH_4_^+^), nitrate‐nitrite nitrogen (S_NO_x_^−^), total phosphorus (S_TP), total organic matter (S_TOM)
Overlying water	Total nitrogen (W_TN), ammonium nitrogen (W_NH_4_^+^), nitrate nitrogen (W_NO_3_^−^), total phosphorus (W_TP), total organic carbon (TOC), Chemical Oxygen Demand (COD), temperature (T), chlorophyll a (Chla), pH, Secchi Disc (SD)
Porewater	Ammonium (P_NH_4_^+^), nitrate nitrogen (P_NO_3_^−^)

Details of the measurement procedures for each parameter can be found in Haller et al. (2011), Tang et al. (2009), Bai et al. (2012). Temperature (T), chlorophyll a (Chla), and pH of the overlying water were measured in situ using an YSI 6600 Multi‐Parameter Water Quality Sonde (YSI), whereas the transparency was determined by a standard Secchi disc (SD) (diameter 20 centimeters) with black and white quadrants (Canfield, Langeland, Linda, & Haller, 1985).

### 2.3 DNA extraction and purification

Total genomic DNA of each sediment sample was extracted using Powersoil DNA extraction kit (Mo bio Laboratories) according to the manufacturer's instructions. The crude DNA extracted was further purified using the PowerClean DNA Clean‐Up Kit (Mo Bio laboratories) for subsequent PCR‐DGGE analysis and Illumina MiSeq sequencing.

### 2.4 Illumina MiSeq sequencing

Based upon the PCR‐DGGE results (see Supplementary Information), Illumina MiSeq sequencing was performed on 24 sediment samples preselected (3 replicates in each sample). PCR amplicon libraries for Illumina MiSeq sequencing were constructed, using bacterial primers 515F (5′‐GTGCCAGCMGCCGCGG‐3′) and 806R (5′‐GGACTACHVGGGTWTCTAAT‐3′) targeting V4 hyper variable regions of bacterial 16S rRNA genes (Caporaso et al., 2012). The conditions for amplification are as follows: 95°C for 2 min; 27 cycles of 95°C for 30 s, 55°C for 30 s, followed by 72°C for 45 s, with a final extension 72°C for 10 min. The PCR products were gel‐purified, using the UltraClean PCR Clean‐Up Kit (Mo Bio laboratories) and quantified, using a Qubit system (Invitrogen). Equimolar amounts of purified amplicons were pooled and stored at −20°C until sequenced. Library construction and sequencing were performed commercially (Beijing Genomics Institute).

### 2.5 Sequences data analyses

Illumina sequence reads were processed using MOTHUR version 1.27.0 (Schloss et al., 2009). Briefly, upon completing sequencing by the Illumina MiSeq platform, the reads from the original DNA fragments were merged, using FLASH (V1.2.7, http://ccb.jhu.edu/software/FLASH/), and quality filtering of reads was performed according to the literature (Caporaso et al., 2011). Chimeric reads were removed by checking against a chimera‐free database of 16S rRNA gene sequences, using UCHIME (DeSantis et al., 2006). Sequences were assigned to the OTUs with a maximum distance of 3%, using MOTHUR (Schloss et al., 2009). Community diversity indices and rarefaction curve of each sample were generated, using the UPARSE pipeline (Edgar, 2013). The RDP classifier was used to assign taxonomic identity to the representative sequence for each OTU.

### 2.6 Statistics analysis

The Trophic Status Indices (*TSI*) (Aizaki, 1981) of all sampling sites were calculated using the measured Chl‐a, W‐TP, W‐TN, COD, and SD by the following expression:

$$TSI(\sum )=\sum_{i=1}^{m}w_{j}\;TSI\left(j\right),$$

where *TSI*(∑) is the completed *TSI*;* w*_*j*_ is the relative weight of *TSI* of the *j* parameter; and *TSI(j)* is *TSI* of the *j* parameter. The mean value of all samples in each region was used to represent the local trophic status which, based upon the value of *TSI* (∑), can be classified as: oligotrophication (0 < *TSI*≤30), mesotrophication (30 < *TSI*≤50), light eutrophication (50 < *TSI*≤60), medium eutrophication (60 < *TSI*≤70), and hypereutrophication (70 < *TSI*≤100) on a scale of 0 to 100.

The OTU lists of samples were submitted to the LEfSe pipeline (LDA Effect size, http://huttenhower.sph.harvard.edu/galaxy/) to identify significant differential features of seasons or sites (Segata et al., 2011). Pearson's correlation analysis (SPSS, v20.0) was performed to determine the links between the bacterial community and the environmental factors (S‐TN, S‐NH_4_, S‐NO_x_, S‐TP, TOM, P‐NH_4_, P‐NO_3_, T, *TSI*). The correlations between microbial OTU composition and the influential factors were determined by Canonical correspondence analysis (CCA) using CANOCO 4.5. The significance tests of Monte Carlo permutations were conducted to construct the appropriate models of the bacteria–environment relationships.

## 3 Results

### 3.1 Physicochemical properties of the samples

**Figure 2:**
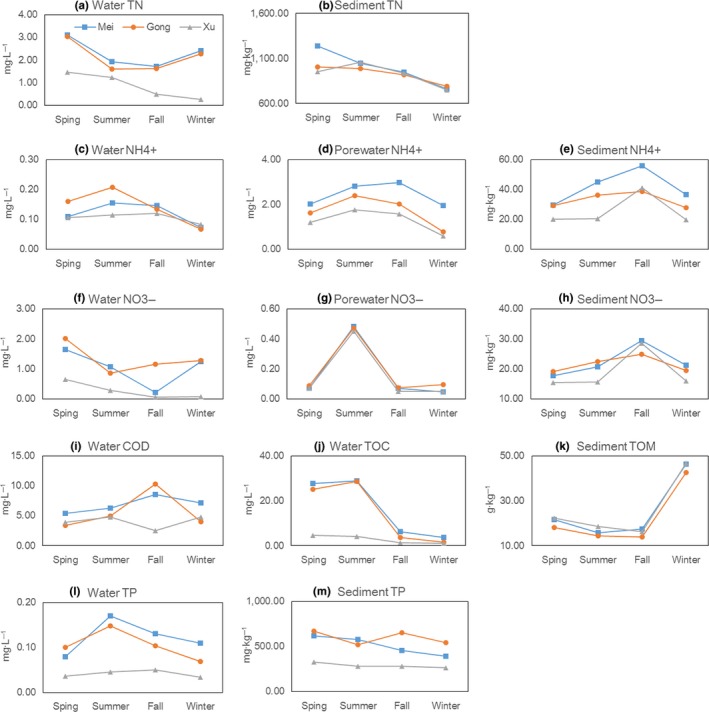
Physiochemical properties of the overlying water, pore water and sediment in different trophic statuses lake in four seasons. A monitoring index concentration of each lake was calculated by taking average value of the monitoring index concentration of the all sample site in this lake

Measured *TSI* in the study area decreased in the direction of A‐1, A‐2, to A‐3 with average values changing from 61.0, 56.3, to 45.3, respectively (Figure 2, Table S1), corresponding to the state of medium eutrophication, light eutrophication, and mesotrophication. For pore water, while the average value of NH_4_^+^‐N decreased in the same direction as *TSI* does (Figure 2d), the concentration of NO_3_^‐^‐N in the research area did not show significant variation (Figure 2g). Similar to the trend of NH_4_^+^‐N in pore water, the contents of NH_4_^+^‐N (Figure 2e) and NO_x_^‐^‐N (Figure 2h) in sediments were reduced with decreasing trophic status. In addition, sediment TOM and TN showed a strong seasonal variation with TOM reaching its maximal level in winter (Figure 2k) and TN in spring (Figure 2b).

### 3.2 Bacterial community structures via Illumina MiSeq sequencing

The similarity of sediment bacterial communities within individual lake regions was first analyzed by PCR‐DGGE. The dendrograms (Figure S1) indicated that the communities in each region can be grouped into 2 defined clusters corresponding to winter and summer. Each cluster can be further divided into two sub‐clusters. Guided by this understanding, we selected two sites in each region, one from each sub‐cluster, and performed further analysis by Illumina MiSeq sequencing.

A total of 1,918,768 high quality sequences (average length 253 bp) were obtained by Illumina MiSeq sequencing at which the rarefaction curves of Shannon diversities approached a plateau, suggesting a complete capture of the bacterial community at each site. Based on a 97% sequence similarity cutoff, these sequences yielded a bacterial OTU number that ranged from 2279 to 4331 (Table S2). Of the three regions, the sites in A‐2 showed the highest diversity in Fall, while the sites in A‐3 reached a peak in the other three seasons. Seasonality‐wise, the lowest diversities were observed in fall and winter.

**Figure 3:**
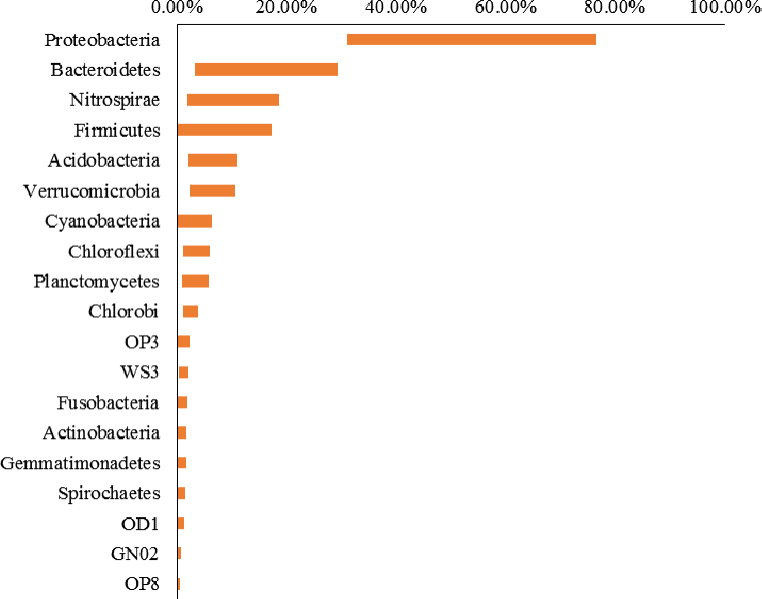
The relative abundance of 19 major bacterial phyla in all sampling sites

**Figure 4:**
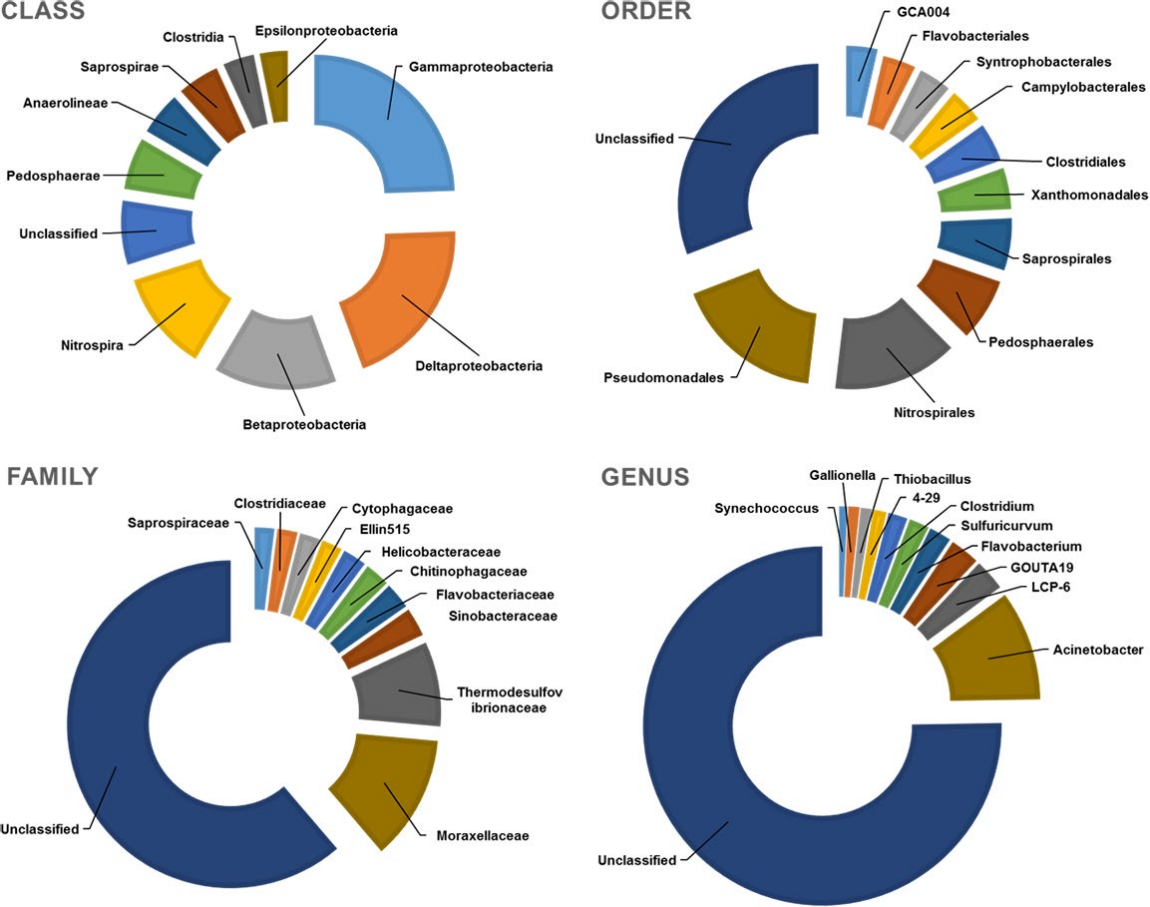
Composition of the top‐10 taxa at the class, order, family, and genus level for all samples

A total of 65 phyla, 221 classes, 436 orders, 624 families, and 864 genera were identified in the study area. Of them, 19 phyla, 52 classes, 79 orders, 70 families, and 49 genera have an abundance greater than 0.5%. Among the 19 major bacterial phyla (Figure 3), *Proteobacteria*, represented by the classes of *gamma‐*,* delta‐*, and *beta‐proteobacteria*, was the most dominant (32.6–76.4% site abundance) in all samples, followed by *Bacteroidetes* (3.3–27.9%), *Nitrospirae* (2.8–18.3%), *Firmicutes* (0.5–14.4%), and *Acidobacteria* (2.2–9.8%) (Table S3 and S4). The top‐10 most abundant phyla accounted for 87.5–97.2% of the total sequences at each site, while the unclassified phyla bacteria in each sample has a relative abundance of 0.6–2.7%. At the levels of class, order, family, and genus, the top‐10 most abundant sequences accounted for approximately 70, 56, 75, and 91% of all sequences, respectively (Figure 4).

Additional taxonomic analyses showed that the most dominant orders were *Pseudomonadales* (0.02–61.9%), *Nitrospirales* (2.8–18.3%), and *Pedosphaerales* (1.6–5.6%) (Table S5), and the most dominant family were *Moraxellaceae* (0.02–61.8%), *Thermodesulfovibrionaceae* (2.4–11.8%), and *Sinobacteraceae* (0.7–4.1%.) (Table S6). At the genera level, *Acinetobacter*, with the relative abundance ranging from 0.01% to 61.8%, was the most dominant. GOUTA19 and LCP‐6 were the other abundant genera and were present in all sediment samples with the relative abundance of 0.5–6.0% and 0.8–5.8%, respectively (Table S7).

### 3.3 The spatial‐temporal distribution of bacterial communities

**Figure 5:**
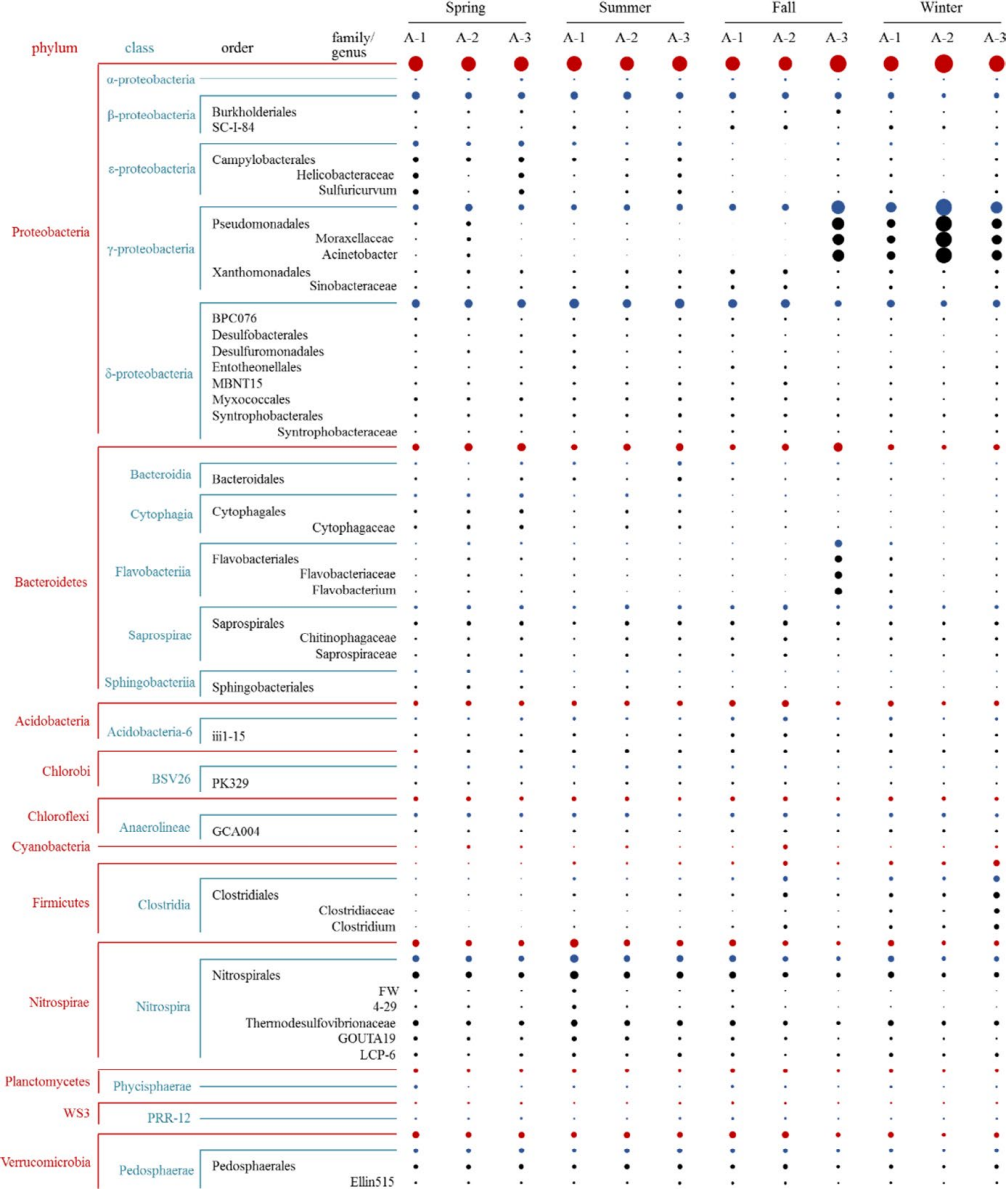
The temporal and spatial variations characteristics of bacterial community structure in different bacterial taxonomical levels (only shown the sequence of bacteria >1% of all sequences). The size of the circle represented the relative abundance of bacteria at each site, and the color of the circle represents bacterial taxonomical levels, red is phylum, blue is class, black is order, family and genus

Spatial‐temporal variation of the bacterial community can be evaluated either by a direct comparison of the relative abundance of individual taxa, or by LeFSe algorithm. Direct comparison found that, for the dominant phyla *Proteobacteria*, its major classes varied greatly with trophic status and seasonal change. For example, *delta‐proteobacteria* and *gamma‐proteobacteria* prevailed in spring/summer and winter, respectively, regardless trophic conditions; meanwhile, the two classes dominated in the eutrophication and mesotrophication lake regions, respectively, but exclusively in Fall (Figure 5 and Table S4). The strongest seasonal dependence may be manifested by *gamma‐proteobacteria* whose abundance showed a greatest decrease from winter and fall to summer and spring (Figure 5). The spatial variation may be exemplified by the behavior of *Planctomycetes*,* Chloroflexi*, and *Bacteroidetes*. For example, whereas *Planctomycetes* and *Chloroflexi* decreased their abundance with lowering trophic status from the eutrophication Meiliang Bay to the mesotrophication Xukou Bay all year long, a reversed pattern was observed for *Bacteroidetes* (Figure 5).

At the genus level, *Acinetobacter* showed the strongest dependence on seasonality (Figure 5) with a significantly higher abundance in the winter than in any other seasons in all but the A‐3 region. Because the magnitude of temporal changes in *Acinetobacter* closely followed those of the upper taxonomy levels, it appears that the genera *Acinetobacter* was the main contributor to the seasonal changes in the lineage of *Moraxellaceae* (family)‐*Pseudomonadales* (order)‐*Gammaproteobacteria* (class). For spatial variation, GOUTA19 stood out to indicate the effect of trophic status as its mean relative abundance decreased with trophic level from > 3.0% in the eutrophic water (A‐1 and A‐2) to <1.9% in the mesotrophic area (A‐3) (Table S7).

**Figure 6:**
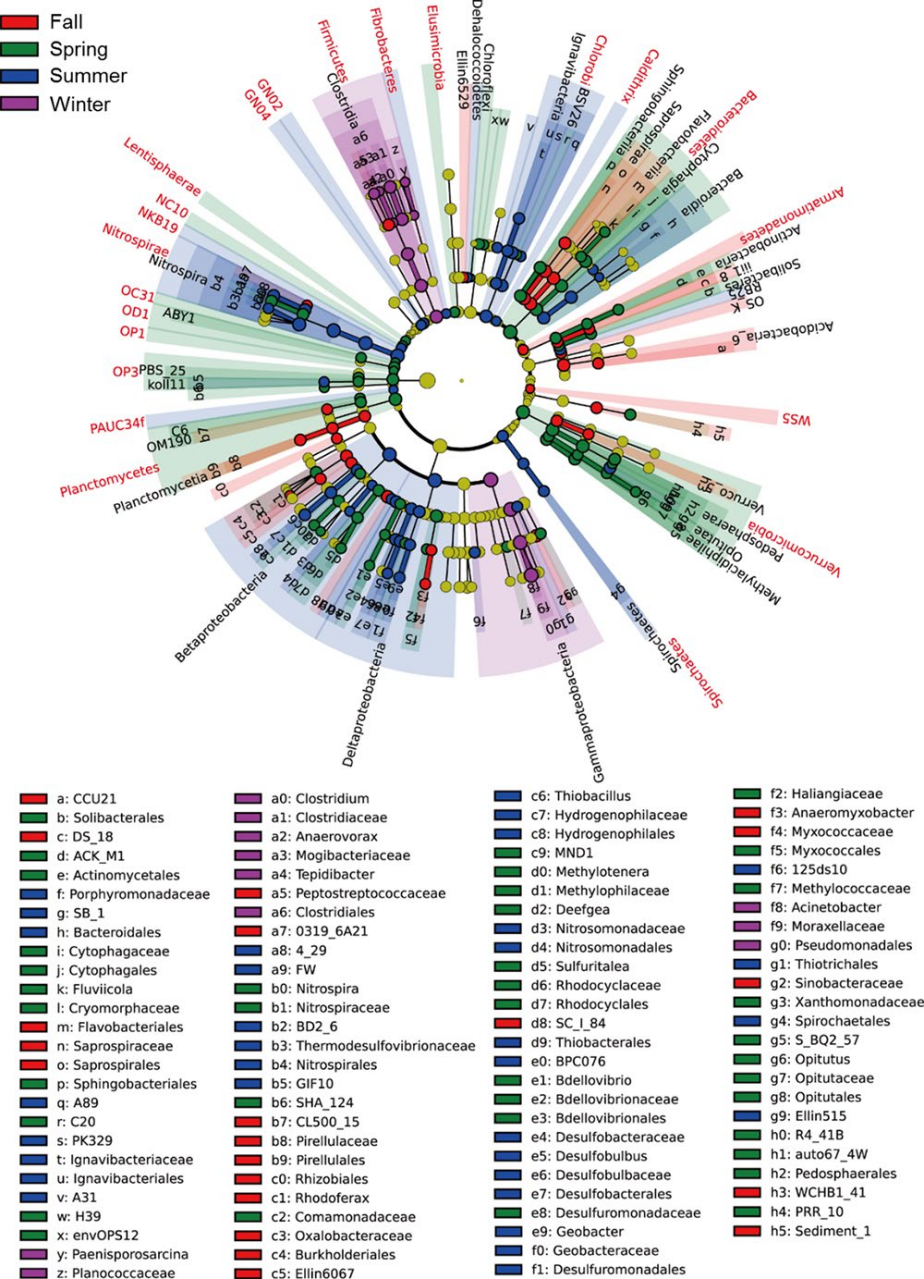
Cladograms indicating the phylogenetic distribution of bacterial lineages associated with the 4 seasons of a year. The phylum, class, order, family, and genus levels are listed in order from inside to outside of the cladogram and the labels for levels of order, family, and genus are abbreviated by a single letter. The green, blue, red, and purple circles represent the bacteria enriched in the sediment of spring, summer, fall, and winter, respectively, whereas the yellow circles represent the taxa with no significant differences between 4 seasons of a year

**Figure 7:**
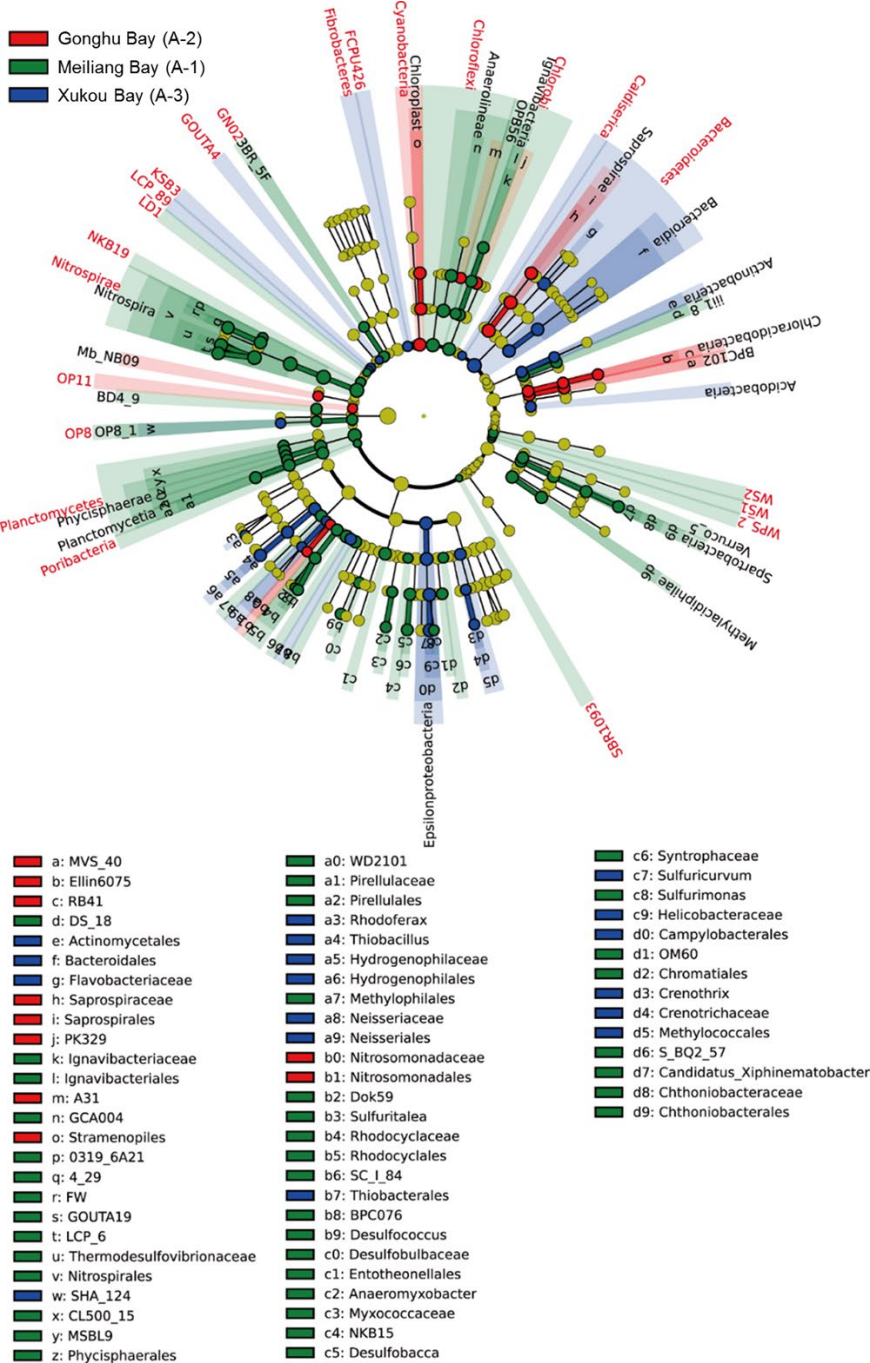
Cladograms indicating the phylogenetic distribution of bacterial lineages associated with the sediments of 3 lake regions. The phylum, class, order, family, and genus levels are listed in order from inside to outside of the cladogram and the labels for levels of order, family, and genus are abbreviated by a single letter. The green, red, and blue circles represent the bacteria enriched in the sediment of Meiliang Bay (A‐1), Gonghu Bay (A‐2), and Xukou Bay (A‐3), respectively, whereas the yellow circles represent the taxa with no significant differences between the sediments of 3 lake regions

For LEfSe analysis, the cladogram showed that, 22 phyla, 32 classes, 42 orders, 39 families, and 19 genera exhibited significant seasonal variation (Figure 6), while 21 phyla, 22 classes, 33 orders, 19 families, and 14 genera had the significant spatial variation (Figure 7). For those that showed the strongest seasonal variation, the majority had the highest abundance in spring and summer (Table 2); for those that showed the strongest spatial variation, a majority had the highest abundance in region A‐1 (Table 3). Some bacterial taxonomy levels (from phylum to family or genus levels) had consistent variation among seasons, such as *Verrucomicrobia*,* Chlorobi*,* Nitrospirae*, and *Firmicutes*, while some bacterial taxonomy levels showed the consistent variation in different regions, such as *Planctomycetes* and *Nitrospirae*. In contrast, the taxonomy levels of *Bacteroidetes* showed the different seasonal and spatial variation.

**Table 2:** The phylogenetic distribution of bacterial lineages associated with the 4 seasons of a year

Taxon	No. of bacterial taxon with significant seasonal variation	Total No. of bacterial taxon in LEfSe analysis	No. of bacterial taxon enriched in the sediment of 4 seasons			
			Spring	Summer	Fall	Winter
Phylum	22	65	10	9	2	1
Class	32	52	13	9	8	2
Order	42	79	14	14	12	2
Family	39	70	16	12	7	4
Genus	19	49	7	5	2	5

**Table 3:** The phylogenetic distribution of bacterial lineages associated with the sediments of 3 lake regions

Taxon	No. of bacterial taxon with significant spatial variation	Total No. of bacterial taxon in LEfSe analysis	No. of bacterial taxon enriched in the sediment of three lake regions		
			Meiliang Bay (A‐1)	Gonghu Bay (A‐2)	Xukou Bay (A‐3)
Phylum	21	65	13	2	6
Class	22	52	13	5	4
Order	33	79	18	7	8
Family	19	70	11	3	5
Genus	14	49	10	0	4

### 3.4 Relationship between bacterial community structure and environmental variable

The overall level of biodiversity in the study area appeared to be correlated positively with the NH_4_^+^‐N in the pore water (*p *< .05) but negatively with NO_x_^−^‐N in the sediment (*p *< .05) (Table 4) as indicated by the bacterial Shannon diversity obtained from Pearson's correlation analysis. For specific taxonomy, a positive correlation between the relative abundance of *Chlorobi*,* Nitrospirae*, and *Beta‐proteobacteria* (*p *<* *.05) and NH_4_^+^‐N in the pore water was matched by a negative one in *gamma‐proteobacteria* (Table 4). Meanwhile, the abundance of *alpha‐proteobacteria* and *delta‐proteobacteria* showed an inverse dependence on NO_x_^−^‐N in the sediments. Finally, the relative abundance of *Chlorobi*,* Chloroflexi*,* Planctomycetes*,* Nitrospirae*, and *Verrucomicrobia*, and 18 bacterial under‐taxonomies all exhibited a significant correlation with *TSI* (Tables 4, 5).

**Table 4:** Statistical analysis of bacterial communities with sediment chemical properties

	T	P_NH_4_^+^	P_NO_3_^−^	S_TN	S_NH_4_^+^	S_NO_x_^−^	S_TP	S_TOM	*TSI*
Shannon diversity	0.32	0.46^a^	0.32	0.30	−0.16	−0.47^a^	0.26	−0.33	0.28
OTU number	0.20	0.19	0.02	0.08	−0.07	−0.18	0.25	−0.30	0.19
*Acidobacteria*	0.03	0.26	0.03	−0.21	0.03	−0.40	0.13	0.07	0.31
*Actinobacteria*	−0.02	0.11	−0.15	−0.06	−0.03	−0.32	0.15	−0.03	0.23
*Bacteroidetes*	0.34	−0.01	−0.06	0.38	0.11	0.35	−0.09	−0.44	−0.41
*Chlorobi*	0.33	0.57^a^	0.40	0.12	0.03	−0.35	0.33	−0.24	0.61^b^
*Chloroflexi*	−0.29	0.28	−0.02	−0.27	0.04	−0.43	0.49^a^	0.27	0.70^b^
*Cyanobacteria*	−0.09	−0.17	−0.23	−0.32	−0.09	−0.33	0.35	−0.00	−0.08
*Firmicutes*	−0.42	−0.22	−0.26	−0.27	−0.14	−0.12	−0.18	0.40	−0.25
*Gemmatimonadetes*	0.12	0.25	0.28	0.07	−0.21	−0.41	0.05	0.01	0.19
*Nitrospirae*	0.23	0.53^a^	0.48^a^	0.19	−0.03	−0.24	0.25	−0.14	0.52^a^
OP3	0.26	0.33	0.25	0.58^a^	−0.16	−0.22	0.41	−0.37	0.39
*Planctomycetes*	0.01	0.44	−0.05	0.06	0.17	−0.30	0.38	0.08	0.66^b^
*Alphaproteobacteria*	0.06	0.05	−0.02	0.11	−0.29	−0.49^a^	0.16	−0.12	0.00
*Betaproteobacteria*	0.56^a^	0.56^a^	0.35	0.61^b^	0.19	0.04	0.03	−0.56^a^	0.26
*Delta proteobacteria*	0.36	0.43	0.43	0.21	−0.15	−0.50^a^	0.12	−0.30	0.42
*Epsilon proteobacteria*	0.12	−0.05	0.06	0.48^a^	−0.20	−0.10	0.03	−0.27	0.14
*Gamma proteobacteria*	−0.41	−0.52^a^	−0.31	−0.43	0.06	0.37	−0.24	0.42	−0.39
*Spirochaetes*	0.43	0.26	0.34	0.30	−0.19	−0.25	−0.17	−0.46^a^	0.04
*Verrucomicrobia*	0.23	0.39	0.09	0.17	−0.04	−0.43	0.35	−0.20	0.45^a^
WS3	0.09	0.26	0.09	0.04	−0.14	−0.35	0.01	0.04	0.12

a Correlation is significant at the 0.05 level.

b Correlation is significant at the 0.01 level.

**Table 5:** The Pearson's correlation analysis of bacteria abundance and trophic status index

Phylum	Taxonomy	*TSI*
*Chlorobi*	*Ignavibacteria* (class)	0.59
	*Ignavibacteriales* (order)	0.59
	*Ignavibacteriaceae* (family)	0.59
*Chloroflexi*	*Anaerolineae* (class)	0.66
	*GCA004* (order)	0.55
*Nitrospirae*	*Nitrospira* (class)	0.52
	*Nitrospirales* (order)	0.52
	*GOUTA19* (genus)	0.72
*Planctomycetes*	*Phycisphaerae* (class)	0.62
	*Phycisphaerales* (order)	0.50
	*Pirellulaceae* (family)	0.52
	*Planctomycetia* (class)	0.51
	*Pirellulales* (order)	0.52
*Verrucomicrobia*	*Methylacidiphilae* (class)	0.61
	*S‐BQ2‐57* (order)	0.61
	*Verruco‐5* (class)	0.54
	*Gamma proteobacteria* (class)	
	*Alteromonadales* (order)	0.51
	*OM60* (family)	0.63

**Figure 8:**
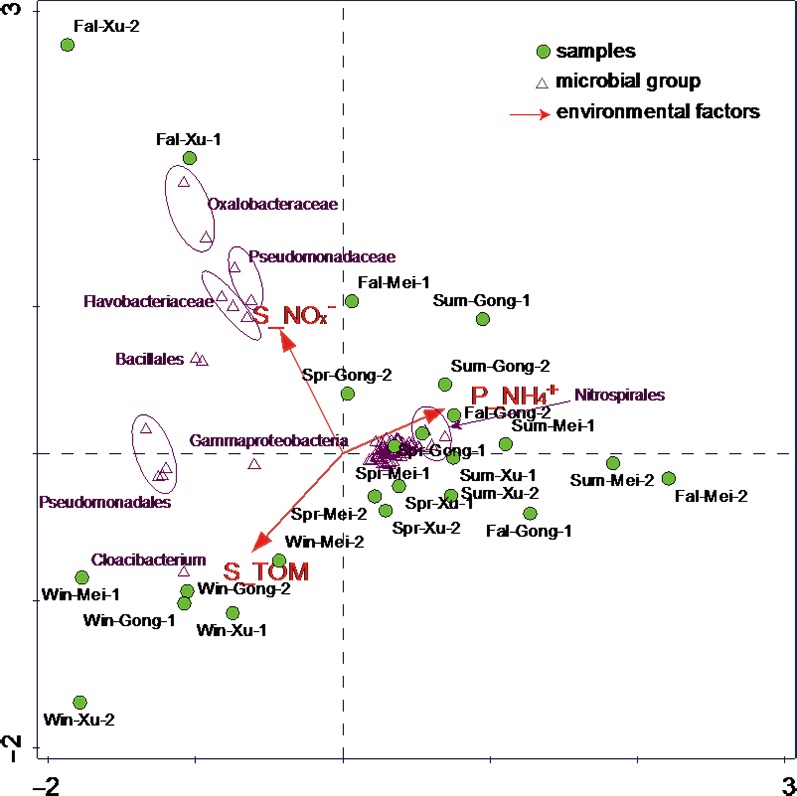
Canonical correspondence analysis (CCA) ordination diagram of bacterial communities associated with environmental variables based on Illumina MiSeq sequencing of the 24 sampling sites in the 4 seasons. The samples were marked as ‘Season‐Lake Region‐Site number’, for example, Spr‐Mei‐1 was one of the sampling site of Meiliang Bay in spring

Lastly, the results of Canonical correspondence analysis (CCA, Figure 8) showed that the differences in bacterial community composition were related to three most important environmental variables, NH_4_^+^‐N in the pore water, as well as NO_x_‐N and TOM in the sediment (*p *< .05, 999 Monte Carlo permutations). Two types of bacterial community distribution were observed corresponding to spring/summer and winter, but individual sites appeared to have a stand‐alone community in fall (Figure 8).

## 4 Discussion

The bacterial OTUs and Shannon diversity obtained in the present study are more than two orders of magnitude and twofold higher than the results acquired via low‐profiling biology techniques for the same/similar eutrophication lakes (Zhao et al., 2013; Szabó et al., 2011), which is similar to that found by the high‐throughput pyrosequencing method (Zhang et al., 2015), suggesting that high‐throughput sequencing techniques are needed to decipher the overall bacterial diversity of lacustrine ecosystems. The relatively simple and stable bacterial community structure in the eutrophication region (A‐1 and A‐2) in comparison to the complexity in the mesotrophic water (A‐3) revealed by this study is consistent with the observations made in Xiamen Sea (Yang et al., 2015), indicating the effect of trophic status on the bacterial diversity may be similar in both marine and freshwater environments.

### 4.1 The characteristics of bacterial community structure

The bacterial communities observed in this study were dominated by *gamm‐*,* delta‐*,* beta‐proteobacteria*, a pattern similar to those found in soils (Liu, Zhang, Zhao, Zhang, & Xie, 2014) and other fresh water lake sediments (Zhang et al., 2015), but distinct from those found in salt water lake sediments (Xiong et al., 2012) and marine coastal waters (Fortunato et al., 2013). It is known that the phylum *Proteobacteria* might be involved in a variety of biogeochemical processes in aquatic ecosystems (Zhang, Zhang, Liu, Xie, & Liu, 2013; Liu et al., 2014). For example, numerous studies, either through conventional approach or high‐throughput method, have shown the predominance of *Proteobacteria* in sediments of various lakes, with a large shift in the composition of major classes and relative proportions (Ye et al., 2009; Haller et al., 2011; Song et al., 2012; Bai et al., 2012). At the class level, both *gamma‐proteobacteria* (Sinkko et al., 2013; Liu et al., 2014) and *delta‐proteobacteria* (Rodionov, Dubchak, Arkin, Alm, & Gelfand, 2004; Lehours, Evans, Bardot, Joblin, & Gerard, 2007) were observed to occur in organic‐rich lacustrine sediments. *Beta‐proteobacteria*, a major class in most of the samples in this study, occurs almost exclusively in freshwater environments (Hempel, Blume, Blindow, & Gross, 2008) and is seen as the most abundant group in the sediments of eutrophication lakes (Bai et al., 2012).

The predominance of *Proteobacteria*'s class and the observed strong correlation between these bacteria and nitrogen conversion (Table 4) in the present study suggest that they were actively involved in the functioning and processes of lake sediment ecosystems (Song et al., 2012). Numerous studies point to a linkage between nitrogen conversion with *Proteobacteria*'s class. For example, Zhang et al. (2013) found *gamma‐proteobacteria* showed a negative correlation with NH_4_^+^‐N; Bai et al. (2012) reported a positive correlation between *beta‐proteobacteria* and the TN. In fact, many nitrogen‐conversion related bacteria, such as ammonia‐oxidizing bacteria, belong to the class *beta‐proteobacteria*. In addition, Zhang et al. (2015) also noted a negative correlation between the content of NO_3_‐N and the relative abundance of *delta‐proteobacteria*. It is thus reasonable to suggest that nitrogen content play an important role in controlling the diversity of bacterial communities in lacustrine environments.

### 4.2 Spatial and seasonal variations in bacterial community structure

The spatial variation of bacterial community is characterized by the dominance of *delta‐proteobacteria* in the eutrophication regions (A‐1 and A‐2) and *gamma‐proteobacteria* in the mesotrophication region (A‐3) in Fall. Such pattern might be due to the regional differences in the sediment organic matters at different trophic levels. In the mesotrophication region, the sediment organic matter is derived mainly from decomposing and dead residues of large vascular plants; in comparison, the sediment organic fraction of the eutrophication regions originated primarily from the organic remains of algae (Qin, Xu, Wu, Luo, & Zhang, 2007). Our results differ from previous research (Shao et al., 2011) where the authors reported that *delta‐proteobacteria* was the prevailing class in the macrophyte‐flourishing areas while *beta‐proteobacteria* was the predominant class in the algae‐blooming areas of the Lake Taihu in the same season. The sources of this discrepancy may be twofold. First of all, the sampling sites of Shao et al. was at the mouth of Meiliang Bay and Xukou Bay where the sediments are frequently resuspended by wind–wave disturbance that disrupts the water–sediment interface, leading to a possible exchange between bacterial communities in the water column and in the surface sediments. Secondly, the conventional DGGE and sequencing methods used in Shao et al. and the clone library coverage may not be sufficient enough to provide a high resolution dissection of the bacterial community, as exemplified by their inability to detect *alpha‐* and *epsilon‐proteobacteria* in the macrophyte‐ and algae‐dominated lake regions (these two classes were detected in all sampling sites by Illumina Miseq Sequencing in the present study).

The reasons for the high abundance of *delta‐proteobacteria* in spring and summer and that of *gamma‐proteobacteria* in winter is not immediately clear. However, this pattern is similar to the observations of Tang et al. (2009, 2010) in their study of the organic aggregate‐associated bacterial community structure in Lake Taihu. Since the waterbody in the lake is shallow (average water depth 1.8 m), the sediment bacterial community may be subject to the same effect of the organic aggregate‐associated bacteria, that is, temperature, NH_4_^+^‐N, and NO_x_^−^‐N.

### 4.3 High abundance bacterial phyla at eutrophic conditions

The high abundance of five phyla at the eutrophication region may be an indication that these microbes have specific nutritional or environmental preference. For example, the observed relation between TP and *Chloroflexi* assemblage (Table 4), along with previous studies in a different lake (Song et al., 2012), may suggest a possible role of phosphorus in promoting the growth of *Chloroflexi*. In addition, it was reported that this phylum was a predominate taxa (57–82%) in the sediment of copper mine (Lucheta, Otero, Macias, & Lambais, 2013). Following this lead, we hypothesize that the high abundance of *Chloroflexi* in region A‐1 may be due to the discharge of phosphorus and heavy metal‐containing industrial wastewater in this area. The observed high TP concentration in the overlying water and sediment in region A‐1(Figure 2) provide additional support for this view point. For *Verrucomicrobia*, the high abundance may be due to the prosthecate morphology of these bacteria which renders a unique ability for nutrient uptake (Zwart et al., 1998). *Verrucomicrobia*, which was able to take advantage of nutrient‐rich environments, had been found in eutrophic ponds and lakes such as those in recreational parks where visitors feed waterfowl (Schlesner, 2004). The positive correlation between NH_4_^+^‐N of the porewater and the abundance of *Chlorobi* is consistent with the data of Edberg, Andersson, and Holmstrom (2012) and suggests these bacteria may have participated in the transformation of NH_4_^+^‐N. Additional factors affecting the presence of *Chlorobi* in mesotrophication regions (A‐3) may include environmental factors because *Chlorobi* are photosynthetic bacteria and hence require the presence of adequate light penetration in water (Vila, Abella, Figueras, & Hurley, 1998). On the contrary, Region A‐3 is teemed with a great many submersed vegetation or aquatic plants. The dense leaves of macrophyte, in particular, can effectively block the transmission of light to the surface of sediments, resulting in an opaque condition that leads to slow growth for *Chlorobi*. *Nitrospirae* is a known significant group related to the nitrite oxidation in freshwater lake sediments (Bartosch, Hartwig, Spieck, & Bock, 2002). Consequently, these bacteria will flourish in high nitrogen condition such as regions A1 and A2. Lastly, positive relation between *Planctomycetes* and eutrophication may be understood from genome analysis (Gloeckner et al., 2003) which revealed the microbes’ ability to derive energy from the degradation of sulfated polysaccharides of algal origin. *Planctomycetes* was present at high levels in diatom blooms (Morris, Longnecker, & Giovannoni, 2006) and in bacterial biofilms on kelp surfaces (Bengtsson & Ovreas, 2010). In fact, the outburst of these bacteria in algae bloom regions suggest that the ecological role of *Planctomycetes* may reside in the degradation of sulfated polysaccharides produced by cyanobacteria.

### 4.4 Factors affecting bacterial community structures

Agreeing with previous studies in other similar eutrophication freshwater lake (Zeng et al., 2008; Dang et al., 2010; Macalady, Mack, Nelson, & Scow, 2000), CCA results from our analyses (Figure 7) showed that pore water NH_4_^+^‐N as well as sediment TOM and NO_x_‐N are likely the dominant environmental factors affecting bacterial community compositions. In fall, the sediments NO_x_^−^‐N in the region A‐3 rises notably, which from 14 mg kg^−1^ in summer to 26 mg kg^−1^ in fall (Figure 2). The high content of NO_x_^−^‐N in region A‐3 in fall directly controls the abundance of bacteria in the taxa of *Oxalobacteraceae*,* Pseudomonadaceae*,* Flavobacteriaceae*, and *Bacillales* (Figure 7) because those bacteria are involved in the transformation of NO_x_‐N (Gaspar, Ferreira, Gonzalez, da Clara, & Santana, 2016; Choi, Lee, & Cha, 2005; Jung et al., 2005; Dodsworth, Hungate, & Hedlund, 2011). In summer, the average value of NH_4_^+^‐N in pore water (2.4 mg L^−1^) is higher than other three seasons (Figure 2), and the higher concentration of NH_4_^+^‐N in pore water promotes the growth of *Nitrospirales* (Figure 7) because the metabolism of this bacteria needs the input of NH_4_^+^‐N (Hamilton et al., 2014), higher abundances of *Nitrospirales* contributed to the higher NH_4_^+^‐N transformation efficiencies (Zhong et al., 2015). The content of TOM is more than 45 g kg^−1^ in winter and less than 20 g kg^−1^ in other three seasons (Figure 2), the increase of TOM in winter directly influence the abundance of *Cloacibacterium* (Figure 7), because these bacteria participate in organic matter degradation, and TOM provide nutrient for the growth of *Cloacibacterium* (Bauer et al., 2006).

## 5 Conclusions

High throughput Illumina MiSeq sequencing method was used to investigate the biodiversity and bacterial community structure in Lake Taihu. More than 1,910,000 sequences were analyzed in the context of changing environmental conditions to evaluate the impact of trophic status on bacterial community, and the results showed significant correlation with trophic status in 5 major phyla and 18 sub‐phylogenetic groups. Findings from this investigation can be summarized as follows:

1. The diversity of bacterial community is inversely related to the trophic levels of water body in most seasons of a year.
2. The bacterial taxa, *delta‐proteobacteria* and *gamma‐proteobacteria*, that dominated, respectively, in the eutrophication and mesotrophication regions showed the strongest seasonal variation.
3. The major environmental factors affecting bacterial community compositions are determined to be NH_4_^+^‐N in porewater as well as TOM and NO_x_‐N in sediments.

## Conflict of Interest

None declared.
