# Supplementary material for: Illumina sequencing‐based analysis of sediment bacteria community in different trophic status freshwater lakes
Source: Microbiologyopen. 2017 Feb 7;6(4):e00450. doi: 10.1002/mbo3.450 (PMC5552931; doi:10.1002/mbo3.450)
Supplement: Supplementary file 1 [file MBO3-6-na-s001.docx]

**Supplementary Information**

**Illumina sequencing-based analysis of sediment bacteria community in different trophic status freshwater lakes**

Yu Wan ^a,b^, Xiaohong Ruan *^, a,b^, Yaping Zhang ^a,b^, Rongfu Li ^a,b^,

^a^ Key Laboratory of Surficial Geochemistry Ministry of Education, Nanjing University, Nanjing 210093, China

^b^ School of Earth Science and Engineering, Nanjing University, Nanjing 210093, China

^[[1]](#footnote-1)^*

**Method**

**Method: PCR amplification and DGGE analysis**

For amplification, DNA samples extracted from three replicate sediments of individual sampling sites were mixed. About 2 ng of the mixture were then used as template for polymerase chain reaction (PCR) amplification of partial 16S rRNA gene. The primers used for PCR analyses were bacterial specific F341 (5’-CCTACGGGAGGCAGCAG-3’) with a 40 bp GC-clamp attached to its 5’ end, and universal R518 (5’-ATTACCGCGGCTGCTGG-3’) (Muyzer et al., 1993).

The DGGE was performed by a DCode^TM^ Universal system (Bio-Rad) using 8% (wt/vol) polyacrylamide gel with a denaturing gradient ranging from 40 to 65% (where 100% is defined as 7 M urea and 40% deionized formamide). A gel documentation system (GelComparⅡ software, Applied Maths) was employed to evaluate the bacterial community in the different sediment samples with Mathematical Averages (UPGMA).

Muyzer, G., Dewaal, E. C., & Uitterlinden, A. G. (1993). Profiling of complex microbial populations by denaturing gradient gel electrophoresis analysis of polymerase chain reaction-amplified genes coding for 16S rRNA. *Applied and Environmental Microbiology*, **59**, 695-700.

**Table S1.** The trophic status index and trophic state evaluation in the research area

| Lake | Trophic Status Index (*TSI*) | | | | | | | Trophic State |
| --- | --- | --- | --- | --- | --- | --- | --- | --- |
|  | Spring |  | Summer |  | Fall |  | Winter |  |
| Meiliang Bay | 60.8 |  | 61.9 |  | 60.8 |  | 60.6 | medium eutrophication |
| Gonghu Bay | 56.5 |  | 56.7 |  | 58.3 |  | 53.6 | light eutrophication |
| Xukou Bay | 48.5 |  | 49.0 |  | 42.2 |  | 41.3 | mesotrophication |

**Table S2.** Numbers of OTUs, and Shannon of 24 sediment samples at a 0.03 cutoff. The samples were marked as ‘Season-Lake Region-Site number’, for example, Spr-Mei-5 was the sample collected from site 5 in the sediment of Meiliang Bay in spring.

| Samples | OTUs | Shannon index |
| --- | --- | --- |
| Spr-Mei-5 | 3272 | 6.47 |
| Spr-Mei-8 | 3491 | 6.76 |
| Spr-Gong-11 | 3413 | 6.64 |
| Spr-Gong-15 | 4052 | 6.85 |
| Spr-Xu-18 | 3874 | 6.97 |
| Spr-Xu-19 | 4096 | 7.10 |
| Sum-Mei-5 | 3339 | 6.71 |
| Sum-Mei-8 | 3114 | 6.63 |
| Sum-Gong-11 | 3423 | 6.91 |
| Sum-Gong-15 | 2921 | 6.75 |
| Sum-Xu-18 | 4157 | 7.04 |
| Sum-Xu-19 | 4331 | 7.13 |
| Fal-Mei-5 | 3205 | 6.65 |
| Fal-Mei-8 | 3427 | 6.70 |
| Fal-Gong-11 | 4112 | 6.73 |
| Fal-Gong-15 | 3675 | 6.74 |
| Fal-Xu-18 | 2708 | 4.74 |
| Fal-Xu-19 | 2925 | 4.66 |
| Win-Mei-5 | 3290 | 6.40 |
| Win-Mei-8 | 3120 | 6.37 |
| Win-Gong-11 | 2279 | 3.76 |
| Win-Gong-15 | 2547 | 4.90 |
| Win-Xu-18 | 3328 | 6.69 |
| Win-Xu-19 | 3163 | 6.48 |

**Table S3.** Abundances of bacterial phylum (%) detected by Illumina sequencing in each sediment samples (largest relative abundance more than 0.5%)

| Taxon/sample lable | Spring | | | | | | | Summer | | | | | | | Fall | | | | | |  | Winter | | | | | |
| --- | --- | --- | --- | --- | --- | --- | --- | --- | --- | --- | --- | --- | --- | --- | --- | --- | --- | --- | --- | --- | --- | --- | --- | --- | --- | --- | --- |
|  | 5# | 8# | 11# | 15# | 18# | 19# |  | 5# | 8# | 11# | 15# | 18# | 19# |  | 5# | 8# | 11# | 15# | 18# | 19# |  | 5# | 8# | 11# | 15# | 18# | 19# |
| *Acidobacteria* | 4.72 | 5.21 | 6.95 | 5.05 | 6.30 | 7.12 |  | 5.10 | 8.08 | 7.58 | 6.19 | 3.76 | 9.13 |  | 7.82 | 9.13 | 9.84 | 9.58 | 2.18 | 3.75 |  | 8.39 | 7.90 | 3.48 | 4.66 | 6.92 | 8.62 |
| *Actinobacteria* | 0.44 | 0.46 | 0.40 | 0.67 | 0.47 | 1.66 |  | 0.50 | 0.61 | 0.52 | 0.43 | 0.31 | 0.48 |  | 0.73 | 0.65 | 1.02 | 0.78 | 0.12 | 0.28 |  | 0.80 | 0.67 | 0.27 | 0.37 | 0.43 | 0.53 |
| *Bacteroidetes* | 6.29 | 10.48 | 7.72 | 22.12 | 11.32 | 15.58 |  | 7.37 | 7.99 | 9.81 | 9.97 | 14.18 | 10.20 |  | 6.49 | 8.51 | 8.99 | 8.69 | 27.94 | 13.11 |  | 6.54 | 5.90 | 3.28 | 4.70 | 8.84 | 6.26 |
| *Chlorobi* | 2.88 | 2.57 | 2.67 | 3.00 | 2.43 | 2.73 |  | 2.95 | 2.47 | 3.37 | 3.51 | 2.70 | 3.35 |  | 3.51 | 3.15 | 2.70 | 3.10 | 1.25 | 1.34 |  | 2.91 | 3.15 | 1.29 | 2.01 | 2.02 | 2.26 |
| *Chloroflexi* | 4.10 | 4.12 | 3.55 | 4.74 | 3.70 | 3.66 |  | 5.18 | 4.05 | 4.35 | 4.25 | 2.25 | 2.66 |  | 4.40 | 4.21 | 4.99 | 4.73 | 1.41 | 1.95 |  | 5.66 | 5.21 | 3.47 | 4.89 | 4.33 | 3.42 |
| *Cyanobacteria* | 0.65 | 0.50 | 1.68 | 2.46 | 1.78 | 1.71 |  | 0.48 | 0.67 | 0.95 | 2.35 | 0.34 | 0.70 |  | 0.99 | 0.63 | 4.28 | 3.66 | 0.37 | 0.46 |  | 0.63 | 0.68 | 0.57 | 1.28 | 2.20 | 2.80 |
| *Firmicutes* | 0.74 | 0.72 | 0.64 | 0.45 | 0.74 | 0.49 |  | 1.19 | 2.34 | 1.23 | 1.76 | 1.78 | 0.83 |  | 1.46 | 2.08 | 4.34 | 5.62 | 1.90 | 2.31 |  | 4.36 | 4.47 | 1.85 | 4.20 | 14.41 | 2.19 |
| *Fusobacteria* | 0.01 | 0.06 | 0.15 | 0.02 | 0.04 | 0.06 |  | 0.02 | 0.58 | 0.01 | 0.01 | 0.81 | 0.01 |  | — | — | 0.01 | — | 0.06 | — |  | — | — | — | — | 0.01 | — |
| GN02 | 0.32 | 0.30 | 0.22 | 0.30 | 0.14 | 0.20 |  | 0.37 | 0.43 | 0.31 | 0.59 | 0.26 | 0.30 |  | 0.27 | 0.31 | 0.20 | 0.27 | 0.06 | 0.14 |  | 0.33 | 0.36 | 0.13 | 0.26 | 0.13 | 0.22 |
| *Gemmatimonadetes* | 0.62 | 1.20 | 1.14 | 0.55 | 0.87 | 1.32 |  | 0.75 | 0.98 | 1.39 | 1.08 | 0.64 | 1.70 |  | 0.74 | 1.34 | 1.05 | 1.03 | 0.25 | 0.39 |  | 1.10 | 1.05 | 0.36 | 0.46 | 0.67 | 1.41 |
| *Nitrospirae* | 9.95 | 9.28 | 6.64 | 10.38 | 7.97 | 6.81 |  | 18.31 | 8.95 | 8.38 | 9.22 | 8.34 | 10.31 |  | 12.69 | 6.27 | 5.56 | 6.42 | 2.89 | 3.09 |  | 9.72 | 9.22 | 2.76 | 6.42 | 9.45 | 3.90 |
| OD1 | 0.34 | 0.57 | 0.39 | 0.74 | 0.40 | 0.91 |  | 0.33 | 0.22 | 0.82 | 0.46 | 0.29 | 0.46 |  | 0.23 | 0.28 | 0.25 | 0.32 | 0.17 | 0.18 |  | 0.31 | 0.20 | 0.10 | 0.18 | 0.43 | 0.29 |
| OP3 | 1.70 | 2.07 | 0.88 | 1.49 | 0.74 | 1.25 |  | 1.03 | 0.44 | 1.21 | 1.33 | 1.28 | 0.61 |  | 0.59 | 0.83 | 0.29 | 0.51 | 0.33 | 0.11 |  | 0.49 | 0.54 | 0.10 | 0.27 | 0.29 | 0.19 |
| OP8 | 0.44 | 0.24 | 0.20 | 0.17 | 0.48 | 0.22 |  | 0.38 | 0.25 | 0.32 | 0.22 | 0.51 | 0.19 |  | 0.49 | 0.47 | 0.19 | 0.19 | 0.29 | 0.14 |  | 0.45 | 0.26 | 0.08 | 0.15 | 0.49 | 0.17 |
| *Planctomycetes* | 4.27 | 5.50 | 3.60 | 2.43 | 2.73 | 3.89 |  | 3.26 | 4.15 | 3.72 | 3.55 | 1.78 | 3.04 |  | 4.63 | 5.23 | 3.67 | 4.37 | 1.05 | 1.70 |  | 4.15 | 4.73 | 1.54 | 2.41 | 2.74 | 3.78 |
| *Proteobacteria* | 49.64 | 40.77 | 49.94 | 32.55 | 45.60 | 35.61 |  | 40.76 | 45.68 | 41.29 | 40.36 | 46.52 | 42.15 |  | 40.19 | 40.91 | 38.99 | 35.31 | 54.48 | 64.94 |  | 43.03 | 42.86 | 76.36 | 60.45 | 36.10 | 51.69 |
| *Spirochaetes* | 0.73 | 0.53 | 0.51 | 0.38 | 1.12 | 0.48 |  | 0.86 | 0.43 | 0.51 | 0.57 | 1.42 | 0.55 |  | 0.73 | 0.49 | 0.62 | 0.53 | 0.45 | 0.22 |  | 0.35 | 0.32 | 0.12 | 0.22 | 0.56 | 0.27 |
| *Verrucomicrobia* | 7.06 | 10.34 | 7.78 | 7.68 | 7.77 | 9.89 |  | 6.08 | 7.64 | 7.98 | 8.41 | 7.10 | 7.50 |  | 8.94 | 9.61 | 8.26 | 9.13 | 2.68 | 3.35 |  | 6.36 | 8.10 | 2.54 | 3.80 | 5.59 | 7.83 |
| WS3 | 1.04 | 1.24 | 1.27 | 0.79 | 1.14 | 2.02 |  | 1.09 | 1.27 | 1.41 | 1.33 | 0.91 | 1.78 |  | 1.48 | 1.88 | 1.07 | 1.64 | 0.44 | 0.92 |  | 1.28 | 1.43 | 0.43 | 0.85 | 1.28 | 1.75 |
| Unclassified | 2.38 | 2.25 | 2.02 | 2.34 | 2.10 | 2.44 |  | 2.39 | 1.46 | 2.66 | 2.47 | 2.44 | 2.03 |  | 2.18 | 2.11 | 2.16 | 2.39 | 0.83 | 0.90 |  | 1.71 | 1.52 | 0.63 | 1.38 | 1.56 | 1.28 |
| Others | 1.65 | 1.60 | 1.64 | 1.70 | 2.15 | 1.95 |  | 1.58 | 1.31 | 2.19 | 1.95 | 2.38 | 2.01 |  | 1.43 | 1.90 | 1.53 | 1.72 | 0.85 | 0.71 |  | 1.43 | 1.41 | 0.63 | 1.06 | 1.55 | 1.15 |

—: not detected.

**Table S4.** Abundances of bacterial classes (%) detected by Illumina sequencing in each sediment samples (largest relative abundance more than 1%)

| Taxon | Spring | | | | | | | Summer | | | | | | | | Fall | | | | | | |  | | | Winter | | | | |
| --- | --- | --- | --- | --- | --- | --- | --- | --- | --- | --- | --- | --- | --- | --- | --- | --- | --- | --- | --- | --- | --- | --- | --- | --- | --- | --- | --- | --- | --- | --- |
|  | 5# | 8# | 11# | 15# | 18# | 19# |  | 5# | 8# | 11# | 15# | 18# | 19# |  | 5# | | 8# | 11# | 15# | 18# | 19# |  | | 5# | 8# | | 11# | 15# | 18# | 19# |
| ***Acidobacteria*** |  |  |  |  |  |  |  |  |  |  |  |  |  |  |  | |  |  |  |  |  |  | |  |  | |  |  |  |  |
| *Acidobacteria-6* | 1.28 | 1.64 | 1.82 | 1.48 | 1.94 | 2.23 |  | 1.47 | 3.49 | 1.71 | 1.64 | 0.78 | 2.67 |  | 3.00 | | 2.98 | 4.26 | 3.73 | 0.57 | 1.13 |  | | 3.29 | 2.94 | | 1.16 | 1.42 | 2.21 | 3.20 |
| *BPC102* | 0.64 | 0.33 | 0.88 | 0.59 | 0.80 | 0.61 |  | 0.60 | 0.58 | 1.10 | 0.94 | 0.64 | 0.72 |  | 0.75 | | 0.90 | 0.92 | 1.08 | 0.41 | 0.65 |  | | 0.73 | 0.67 | | 0.63 | 0.86 | 1.15 | 0.68 |
| *RB25* | 0.63 | 0.81 | 1.04 | 0.43 | 1.07 | 0.89 |  | 0.77 | 0.76 | 1.06 | 0.76 | 0.78 | 1.36 |  | 0.81 | | 0.98 | 0.62 | 0.69 | 0.30 | 0.35 |  | | 0.68 | 0.64 | | 0.19 | 0.25 | 0.68 | 0.78 |
| *iii1-8* | 0.40 | 0.39 | 0.54 | 0.26 | 0.43 | 0.53 |  | 0.50 | 0.73 | 0.52 | 0.42 | 0.21 | 0.63 |  | 0.89 | | 0.90 | 1.02 | 0.91 | 0.16 | 0.30 |  | | 0.93 | 0.78 | | 0.32 | 0.36 | 0.48 | 0.88 |
| ***Actinobacteria*** |  |  |  |  |  |  |  |  |  |  |  |  |  |  |  | |  |  |  |  |  |  | |  |  | |  |  |  |  |
| *Actinobacteria* | 0.18 | 0.24 | 0.13 | 0.54 | 0.14 | 1.18 |  | 0.18 | 0.17 | 0.18 | 0.20 | 0.08 | 0.14 |  | 0.19 | | 0.14 | 0.16 | 0.14 | 0.04 | 0.10 |  | | 0.27 | 0.24 | | 0.06 | 0.14 | 0.09 | 0.09 |
| ***Bacteroidetes*** |  |  |  |  |  |  |  |  |  |  |  |  |  |  |  | |  |  |  |  |  |  | |  |  | |  |  |  |  |
| *Bacteroidia* | 1.31 | 1.25 | 0.50 | 1.44 | 2.99 | 1.23 |  | 1.74 | 2.15 | 0.91 | 0.93 | 6.78 | 0.71 |  | 1.53 | | 0.74 | 0.89 | 0.50 | 1.23 | 0.53 |  | | 1.03 | 0.69 | | 0.20 | 0.41 | 2.11 | 0.36 |
| *Cytophagia* | 0.85 | 3.11 | 1.79 | 5.29 | 2.16 | 4.91 |  | 0.85 | 0.98 | 2.27 | 1.93 | 2.27 | 1.92 |  | 0.43 | | 0.95 | 1.06 | 0.88 | 0.65 | 0.27 |  | | 0.52 | 0.50 | | 0.30 | 0.36 | 1.37 | 0.72 |
| *Flavobacteriia* | 0.54 | 1.14 | 0.63 | 3.47 | 0.83 | 1.79 |  | 1.36 | 1.00 | 0.55 | 0.94 | 0.77 | 0.58 |  | 0.39 | | 0.81 | 0.22 | 0.29 | 23.77 | 9.46 |  | | 0.32 | 0.33 | | 0.18 | 0.20 | 0.65 | 0.23 |
| *Saprospirae* | 1.84 | 2.23 | 3.11 | 4.67 | 3.67 | 4.43 |  | 1.66 | 2.63 | 4.05 | 3.96 | 2.26 | 5.05 |  | 2.73 | | 4.49 | 5.61 | 5.57 | 1.64 | 2.26 |  | | 3.13 | 2.99 | | 2.09 | 2.90 | 3.76 | 3.73 |
| *Sphingobacteriia* | 0.71 | 1.85 | 1.08 | 5.64 | 0.83 | 1.89 |  | 0.70 | 0.71 | 0.99 | 1.40 | 0.64 | 1.25 |  | 0.55 | | 1.08 | 0.52 | 0.92 | 0.30 | 0.39 |  | | 0.64 | 0.82 | | 0.28 | 0.59 | 0.57 | 0.84 |
| ***Chlorobi*** |  |  |  |  |  |  |  |  |  |  |  |  |  |  |  | |  |  |  |  |  |  | |  |  | |  |  |  |  |
| *BSV26* | 1.42 | 1.73 | 1.83 | 1.84 | 1.38 | 2.02 |  | 1.48 | 1.37 | 2.12 | 1.95 | 1.35 | 2.29 |  | 1.68 | | 1.95 | 1.43 | 1.83 | 0.64 | 0.90 |  | | 1.37 | 1.41 | | 0.61 | 1.06 | 1.14 | 1.54 |
| *Ignavibacteria* | 0.90 | 0.43 | 0.47 | 0.71 | 0.65 | 0.42 |  | 1.02 | 0.52 | 0.76 | 0.95 | 0.97 | 0.61 |  | 1.02 | | 0.77 | 0.56 | 0.63 | 0.37 | 0.30 |  | | 0.92 | 0.81 | | 0.41 | 0.60 | 0.48 | 0.36 |
| ***Chloroflexi*** |  |  |  |  |  |  |  |  |  |  |  |  |  |  |  | |  |  |  |  |  |  | |  |  | |  |  |  |  |
| *Anaerolineae* | 3.59 | 3.66 | 3.04 | 4.40 | 3.26 | 3.10 |  | 4.65 | 3.42 | 3.82 | 3.73 | 1.84 | 1.86 |  | 3.84 | | 3.36 | 4.22 | 4.04 | 1.27 | 1.64 |  | | 5.17 | 4.73 | | 3.24 | 4.49 | 3.97 | 2.77 |
| ***Cyanobacteria*** |  |  |  |  |  |  |  |  |  |  |  |  |  |  |  | |  |  |  |  |  |  | |  |  | |  |  |  |  |
| *Chloroplast* | 0.33 | 0.32 | 1.21 | 2.18 | 0.23 | 0.73 |  | 0.29 | 0.25 | 0.64 | 1.96 | 0.05 | 0.13 |  | 0.30 | | 0.09 | 1.79 | 1.83 | 0.04 | 0.02 |  | | 0.09 | 0.03 | | 0.12 | 0.61 | 0.11 | 0.09 |
| *Synechococcophycideae* | 0.15 | 0.06 | 0.30 | 0.06 | 1.46 | 0.75 |  | 0.09 | 0.33 | 0.19 | 0.17 | 0.24 | 0.51 |  | 0.46 | | 0.46 | 2.24 | 1.67 | 0.31 | 0.41 |  | | 0.40 | 0.60 | | 0.43 | 0.61 | 2.05 | 2.67 |
| ***Firmicutes*** |  |  |  |  |  |  |  |  |  |  |  |  |  |  |  | |  |  |  |  |  |  | |  |  | |  |  |  |  |
| *Clostridia* | 0.73 | 0.69 | 0.64 | 0.43 | 0.73 | 0.46 |  | 1.19 | 2.26 | 1.22 | 1.74 | 1.76 | 0.83 |  | 1.40 | | 2.05 | 4.29 | 5.60 | 1.35 | 1.78 |  | | 4.27 | 4.46 | | 1.83 | 3.73 | 14.38 | 2.18 |
| ***Gemmatimonadetes*** |  |  |  |  |  |  |  |  |  |  |  |  |  |  |  | |  |  |  |  |  |  | |  |  | |  |  |  |  |
| *Gemm-1* | 0.57 | 0.97 | 1.01 | 0.40 | 0.79 | 1.01 |  | 0.68 | 0.90 | 1.19 | 0.91 | 0.58 | 1.53 |  | 0.69 | | 1.20 | 0.95 | 0.96 | 0.23 | 0.36 |  | | 1.06 | 0.99 | | 0.33 | 0.43 | 0.62 | 1.33 |
| ***Nitrospirae*** |  |  |  |  |  |  |  |  |  |  |  |  |  |  |  | |  |  |  |  |  |  | |  |  | |  |  |  |  |
| *Nitrospira* | 9.95 | 9.28 | 6.64 | 10.38 | 7.97 | 6.81 |  | 18.31 | 8.95 | 8.38 | 9.22 | 8.34 | 10.31 |  | 12.69 | | 6.27 | 5.56 | 6.42 | 2.89 | 3.09 |  | | 9.72 | 9.22 | | 2.76 | 6.42 | 9.45 | 3.90 |
| ***Planctomycetes*** |  |  |  |  |  |  |  |  |  |  |  |  |  |  |  | |  |  |  |  |  |  | |  |  | |  |  |  |  |
| *Phycisphaerae* | 2.17 | 2.34 | 0.96 | 0.57 | 0.99 | 0.94 |  | 1.24 | 1.76 | 1.30 | 1.16 | 0.71 | 1.18 |  | 2.13 | | 2.27 | 1.21 | 1.45 | 0.44 | 0.80 |  | | 2.03 | 2.11 | | 0.40 | 0.86 | 1.03 | 1.48 |
| *Planctomycetia* | 0.85 | 0.78 | 0.63 | 0.63 | 0.59 | 0.74 |  | 0.63 | 1.09 | 0.48 | 0.71 | 0.26 | 0.50 |  | 1.24 | | 1.27 | 1.24 | 1.46 | 0.22 | 0.40 |  | | 1.24 | 1.45 | | 0.75 | 1.00 | 0.85 | 1.07 |
| ***Proteobacteria*** |  |  |  |  |  |  |  |  |  |  |  |  |  |  |  | |  |  |  |  |  |  | |  |  | |  |  |  |  |
| *Alphaproteobacteria* | 0.79 | 1.67 | 1.53 | 1.63 | 1.46 | 2.50 |  | 0.67 | 1.40 | 1.68 | 1.37 | 0.85 | 2.43 |  | 0.98 | | 2.22 | 1.72 | 1.90 | 0.89 | 0.73 |  | | 1.21 | 1.34 | | 0.79 | 1.25 | 1.59 | 1.45 |
| *Betaproteobacteria* | 13.69 | 12.51 | 9.57 | 9.32 | 11.87 | 10.44 |  | 12.59 | 11.27 | 12.85 | 12.84 | 12.29 | 8.99 |  | 10.42 | | 11.32 | 9.57 | 8.35 | 16.25 | 4.37 |  | | 8.95 | 8.47 | | 3.48 | 5.22 | 7.92 | 5.51 |
| *Deltaproteobacteria* | 14.83 | 14.13 | 15.91 | 11.70 | 17.15 | 13.63 |  | 16.37 | 17.73 | 14.76 | 14.94 | 17.75 | 18.31 |  | 17.17 | | 14.57 | 16.67 | 15.00 | 7.25 | 5.99 |  | | 14.15 | 14.15 | | 6.07 | 9.70 | 14.93 | 11.41 |
| *Epsilonproteobacteria* | 13.51 | 2.72 | 7.53 | 0.66 | 5.87 | 0.54 |  | 1.06 | 5.54 | 1.13 | 1.59 | 7.55 | 0.24 |  | 0.38 | | 0.39 | 0.04 | 0.02 | 2.57 | 0.05 |  | | 0.10 | 1.13 | | 0.05 | 0.31 | 0.59 | 0.24 |
| *Gammaproteobacteria* | 6.18 | 8.79 | 14.27 | 8.53 | 8.26 | 7.58 |  | 5.44 | 8.47 | 9.76 | 8.31 | 6.75 | 11.17 |  | 8.47 | | 11.58 | 9.91 | 8.84 | 27.11 | 53.54 |  | | 18.02 | 16.78 | | 65.65 | 43.42 | 10.42 | 32.46 |
| ***Spirochaetes*** |  |  |  |  |  |  |  |  |  |  |  |  |  |  |  | |  |  |  |  |  |  | |  |  | |  |  |  |  |
| *Spirochaetes* | 0.50 | 0.39 | 0.34 | 0.19 | 0.73 | 0.29 |  | 0.66 | 0.32 | 0.32 | 0.37 | 1.07 | 0.38 |  | 0.51 | | 0.28 | 0.34 | 0.30 | 0.31 | 0.13 |  | | 0.25 | 0.23 | | 0.06 | 0.12 | 0.36 | 0.17 |
| ***Verrucomicrobia*** |  |  |  |  |  |  |  |  |  |  |  |  |  |  |  | |  |  |  |  |  |  | |  |  | |  |  |  |  |
| *Methylacidiphilae* | 1.19 | 2.76 | 1.43 | 1.39 | 0.29 | 1.27 |  | 0.55 | 0.71 | 1.29 | 1.48 | 0.22 | 0.56 |  | 2.02 | | 1.06 | 1.04 | 1.18 | 0.15 | 0.21 |  | | 0.91 | 1.41 | | 0.21 | 0.33 | 0.20 | 0.63 |
| *Pedosphaerae* | 3.86 | 5.58 | 4.78 | 4.81 | 5.50 | 5.83 |  | 3.70 | 3.38 | 5.04 | 4.93 | 5.55 | 4.97 |  | 3.64 | | 5.57 | 4.85 | 5.11 | 1.83 | 2.15 |  | | 3.45 | 3.89 | | 1.65 | 2.20 | 3.62 | 4.25 |
| *Spartobacteria* | 0.42 | 0.39 | 0.49 | 0.28 | 0.47 | 0.67 |  | 0.27 | 2.26 | 0.30 | 0.63 | 0.17 | 0.68 |  | 1.66 | | 1.14 | 0.70 | 1.04 | 0.22 | 0.48 |  | | 0.88 | 1.82 | | 0.29 | 0.56 | 0.60 | 1.89 |
| *Verruco-5* | 1.13 | 0.91 | 0.63 | 0.41 | 0.70 | 0.98 |  | 0.95 | 0.67 | 0.83 | 0.85 | 0.72 | 0.89 |  | 0.90 | | 1.16 | 1.09 | 1.08 | 0.22 | 0.27 |  | | 0.63 | 0.64 | | 0.19 | 0.34 | 0.34 | 0.65 |
| ***WS3*** |  |  |  |  |  |  |  |  |  |  |  |  |  |  |  | |  |  |  |  |  |  | |  |  | |  |  |  |  |
| *PRR-12* | 1.04 | 1.24 | 1.27 | 0.79 | 1.14 | 2.02 |  | 1.09 | 1.27 | 1.41 | 1.33 | 0.91 | 1.78 |  | 1.48 | | 1.88 | 1.07 | 1.64 | 0.44 | 0.92 |  | | 1.28 | 1.43 | | 0.43 | 0.85 | 1.28 | 1.75 |

**Table S5.** Abundances of bacterial order (%) detected by Illumina sequencing in each sediment samples (largest relative abundance more than 1%)

| Taxon | Spring | | | | | | | Summer | | | | | | | | Fall | | | | | | |  | | | Winter | | | | |
| --- | --- | --- | --- | --- | --- | --- | --- | --- | --- | --- | --- | --- | --- | --- | --- | --- | --- | --- | --- | --- | --- | --- | --- | --- | --- | --- | --- | --- | --- | --- |
|  | 5# | 8# | 11# | 15# | 18# | 19# |  | 5# | 8# | 11# | 15# | 18# | 19# |  | 5# | | 8# | 11# | 15# | 18# | 19# |  | | 5# | 8# | | 11# | 15# | 18# | 19# |
| ***Acidobacteria*** |  |  |  |  |  |  |  |  |  |  |  |  |  |  |  | |  |  |  |  |  |  | |  |  | |  |  |  |  |
| *CCU21* | 0.17 | 0.21 | 0.36 | 0.10 | 0.33 | 0.40 |  | 0.19 | 0.58 | 0.25 | 0.22 | 0.10 | 0.34 |  | 0.45 | | 0.53 | 1.06 | 0.78 | 0.06 | 0.24 |  | | 0.43 | 0.41 | | 0.21 | 0.24 | 0.28 | 0.62 |
| *iii1-15* | 1.10 | 1.42 | 1.45 | 1.37 | 1.60 | 1.82 |  | 1.25 | 2.87 | 1.46 | 1.41 | 0.67 | 2.32 |  | 2.52 | | 2.44 | 3.20 | 2.93 | 0.50 | 0.88 |  | | 2.84 | 2.48 | | 0.94 | 1.15 | 1.92 | 2.56 |
| ***Actinobacteria*** |  |  |  |  |  |  |  |  |  |  |  |  |  |  |  | |  |  |  |  |  |  | |  |  | |  |  |  |  |
| *Actinomycetales* | 0.11 | 0.23 | 0.12 | 0.54 | 0.10 | 1.18 |  | 0.14 | 0.12 | 0.18 | 0.17 | 0.05 | 0.10 |  | 0.06 | | 0.09 | 0.11 | 0.09 | 0.02 | 0.03 |  | | 0.14 | 0.15 | | 0.03 | 0.03 | 0.03 | 0.06 |
| ***Bacteroidetes*** |  |  |  |  |  |  |  |  |  |  |  |  |  |  |  | |  |  |  |  |  |  | |  |  | |  |  |  |  |
| *Bacteroidales* | 1.31 | 1.25 | 0.50 | 1.44 | 2.99 | 1.23 |  | 1.74 | 2.15 | 0.91 | 0.93 | 6.78 | 0.71 |  | 1.53 | | 0.74 | 0.89 | 0.50 | 1.23 | 0.53 |  | | 1.03 | 0.69 | | 0.20 | 0.41 | 2.11 | 0.36 |
| *Cytophagales* | 0.85 | 3.11 | 1.79 | 5.29 | 2.16 | 4.91 |  | 0.85 | 0.98 | 2.27 | 1.93 | 2.27 | 1.92 |  | 0.43 | | 0.95 | 1.06 | 0.88 | 0.65 | 0.27 |  | | 0.52 | 0.50 | | 0.30 | 0.36 | 1.37 | 0.72 |
| *Flavobacteriales* | 0.54 | 1.14 | 0.63 | 3.47 | 0.83 | 1.79 |  | 1.36 | 1.00 | 0.55 | 0.94 | 0.77 | 0.58 |  | 0.39 | | 0.81 | 0.22 | 0.29 | 23.77 | 9.46 |  | | 0.32 | 0.33 | | 0.18 | 0.20 | 0.65 | 0.23 |
| *Saprospirales* | 1.84 | 2.23 | 3.11 | 4.67 | 3.67 | 4.43 |  | 1.66 | 2.63 | 4.05 | 3.96 | 2.26 | 5.05 |  | 2.73 | | 4.49 | 5.61 | 5.57 | 1.64 | 2.26 |  | | 3.13 | 2.99 | | 2.09 | 2.90 | 3.76 | 3.73 |
| *Sphingobacteriales* | 0.71 | 1.85 | 1.08 | 5.64 | 0.83 | 1.89 |  | 0.70 | 0.71 | 0.99 | 1.40 | 0.64 | 1.25 |  | 0.55 | | 1.08 | 0.52 | 0.92 | 0.30 | 0.39 |  | | 0.64 | 0.82 | | 0.28 | 0.59 | 0.57 | 0.84 |
| ***Chlorobi*** |  |  |  |  |  |  |  |  |  |  |  |  |  |  |  | |  |  |  |  |  |  | |  |  | |  |  |  |  |
| *PK329* | 1.21 | 1.20 | 1.54 | 1.43 | 1.14 | 1.35 |  | 1.20 | 1.13 | 1.66 | 1.57 | 1.01 | 1.61 |  | 1.45 | | 1.55 | 1.22 | 1.60 | 0.52 | 0.78 |  | | 1.22 | 1.22 | | 0.55 | 0.98 | 0.93 | 1.15 |
| *Ignavibacteriales* | 0.90 | 0.43 | 0.47 | 0.71 | 0.65 | 0.42 |  | 1.02 | 0.52 | 0.76 | 0.95 | 0.97 | 0.61 |  | 1.02 | | 0.77 | 0.56 | 0.63 | 0.37 | 0.30 |  | | 0.92 | 0.81 | | 0.41 | 0.60 | 0.48 | 0.36 |
| ***Chloroflexi*** |  |  |  |  |  |  |  |  |  |  |  |  |  |  |  | |  |  |  |  |  |  | |  |  | |  |  |  |  |
| *GCA004* | 2.05 | 1.49 | 1.71 | 1.62 | 1.67 | 1.31 |  | 2.68 | 2.22 | 2.08 | 2.41 | 0.77 | 0.93 |  | 2.26 | | 1.79 | 2.58 | 2.41 | 0.61 | 0.99 |  | | 3.54 | 3.11 | | 2.45 | 3.37 | 2.31 | 1.74 |
| ***Cyanobacteria*** |  |  |  |  |  |  |  |  |  |  |  |  |  |  |  | |  |  |  |  |  |  | |  |  | |  |  |  |  |
| *Stramenopiles* | 0.32 | 0.32 | 1.19 | 2.16 | 0.23 | 0.72 |  | 0.29 | 0.25 | 0.64 | 1.95 | 0.04 | 0.13 |  | 0.30 | | 0.09 | 1.74 | 1.83 | 0.03 | 0.02 |  | | 0.09 | 0.03 | | 0.12 | 0.61 | 0.10 | 0.09 |
| *Synechococcales* | 0.14 | 0.06 | 0.30 | 0.06 | 1.46 | 0.75 |  | 0.09 | 0.33 | 0.19 | 0.17 | 0.24 | 0.51 |  | 0.45 | | 0.46 | 2.23 | 1.66 | 0.31 | 0.41 |  | | 0.39 | 0.60 | | 0.43 | 0.61 | 2.02 | 2.67 |
| ***Firmicutes*** |  |  |  |  |  |  |  |  |  |  |  |  |  |  |  | |  |  |  |  |  |  | |  |  | |  |  |  |  |
| *Clostridiales* | 0.71 | 0.69 | 0.63 | 0.43 | 0.71 | 0.45 |  | 1.18 | 2.26 | 1.21 | 1.74 | 1.75 | 0.82 |  | 1.39 | | 2.05 | 4.29 | 5.60 | 1.34 | 1.76 |  | | 4.27 | 4.45 | | 1.83 | 3.73 | 14.37 | 2.18 |
| ***Nitrospirae*** |  |  |  |  |  |  |  |  |  |  |  |  |  |  |  | |  |  |  |  |  |  | |  |  | |  |  |  |  |
| *Nitrospirales* | 9.95 | 9.28 | 6.64 | 10.38 | 7.97 | 6.81 |  | 18.31 | 8.95 | 8.38 | 9.22 | 8.34 | 10.31 |  | 12.69 | | 6.27 | 5.56 | 6.42 | 2.89 | 3.09 |  | | 9.72 | 9.22 | | 2.76 | 6.42 | 9.45 | 3.90 |
| ***Planctomycetes*** |  |  |  |  |  |  |  |  |  |  |  |  |  |  |  | |  |  |  |  |  |  | |  |  | |  |  |  |  |
| *MSBL9* | 1.10 | 1.04 | 0.16 | 0.24 | 0.28 | 0.11 |  | 0.52 | 0.74 | 0.28 | 0.22 | 0.23 | 0.11 |  | 0.85 | | 0.69 | 0.18 | 0.21 | 0.10 | 0.09 |  | | 0.57 | 0.76 | | 0.04 | 0.13 | 0.20 | 0.07 |
| *Pirellulales* | 0.73 | 0.65 | 0.52 | 0.44 | 0.46 | 0.53 |  | 0.56 | 0.96 | 0.40 | 0.58 | 0.22 | 0.37 |  | 1.13 | | 1.09 | 1.06 | 1.25 | 0.20 | 0.32 |  | | 1.09 | 1.23 | | 0.65 | 0.89 | 0.71 | 0.92 |
| ***Betaproteobacteria*** |  |  |  |  |  |  |  |  |  |  |  |  |  |  |  | |  |  |  |  |  |  | |  |  | |  |  |  |  |
| *Burkholderiales* | 0.67 | 1.72 | 1.48 | 1.88 | 1.61 | 2.37 |  | 0.93 | 1.12 | 1.32 | 1.08 | 0.99 | 1.25 |  | 1.00 | | 1.10 | 1.28 | 0.97 | 9.04 | 1.56 |  | | 1.13 | 0.77 | | 0.51 | 0.62 | 1.24 | 0.85 |
| *Ellin6067* | 0.41 | 0.58 | 0.93 | 0.13 | 0.51 | 0.57 |  | 0.44 | 0.80 | 0.92 | 0.64 | 0.32 | 0.93 |  | 0.78 | | 1.00 | 1.00 | 0.94 | 0.26 | 0.21 |  | | 0.53 | 0.63 | | 0.23 | 0.29 | 0.54 | 0.51 |
| *Gallionellales* | 1.95 | 1.89 | 1.00 | 1.22 | 1.20 | 0.69 |  | 1.82 | 1.12 | 2.85 | 2.45 | 1.36 | 0.51 |  | 0.63 | | 0.59 | 0.20 | 0.33 | 0.24 | 0.03 |  | | 0.36 | 0.41 | | 0.11 | 0.38 | 0.21 | 0.11 |
| *Hydrogenophilales* | 0.92 | 1.22 | 0.37 | 0.43 | 1.71 | 0.47 |  | 1.49 | 0.75 | 0.74 | 0.86 | 2.45 | 0.42 |  | 0.86 | | 0.85 | 0.20 | 0.30 | 4.56 | 1.02 |  | | 0.27 | 0.59 | | 0.05 | 0.19 | 0.80 | 0.28 |
| *MND1* | 0.12 | 0.39 | 0.39 | 0.22 | 0.25 | 0.70 |  | 0.12 | 0.25 | 0.41 | 0.34 | 0.10 | 1.01 |  | 0.07 | | 0.24 | 0.25 | 0.36 | 0.06 | 0.20 |  | | 0.07 | 0.14 | | 0.10 | 0.16 | 0.23 | 0.46 |
| *Methylophilales* | 1.38 | 0.64 | 0.28 | 1.03 | 0.77 | 0.86 |  | 1.46 | 0.44 | 0.28 | 0.61 | 0.80 | 0.18 |  | 0.76 | | 0.37 | 0.08 | 0.15 | 0.19 | 0.06 |  | | 0.24 | 0.26 | | 0.01 | 0.16 | 0.41 | 0.09 |
| *Rhodocyclales* | 1.09 | 1.22 | 0.81 | 0.67 | 0.81 | 0.68 |  | 0.93 | 1.41 | 0.57 | 0.91 | 0.96 | 0.23 |  | 0.45 | | 0.41 | 0.36 | 0.24 | 0.25 | 0.05 |  | | 0.27 | 0.24 | | 0.29 | 0.14 | 0.32 | 0.16 |
| *SBla14* | 1.99 | 0.56 | 0.29 | 0.79 | 0.57 | 0.37 |  | 0.91 | 0.47 | 0.71 | 0.98 | 0.75 | 0.10 |  | 0.32 | | 0.14 | 0.14 | 0.07 | 0.14 | 0.02 |  | | 0.13 | 0.12 | | 0.03 | 0.18 | 0.19 | 0.01 |
| *SC-I-84* | 1.37 | 0.77 | 1.59 | 0.53 | 1.02 | 0.89 |  | 1.10 | 2.40 | 1.86 | 1.20 | 0.55 | 1.78 |  | 3.25 | | 3.93 | 4.07 | 3.17 | 0.32 | 0.70 |  | | 3.98 | 3.34 | | 1.42 | 1.92 | 1.76 | 1.73 |
| *Thiobacterales* | 0.15 | 0.34 | 0.15 | 0.16 | 1.21 | 0.12 |  | 0.23 | 0.14 | 0.16 | 0.19 | 1.77 | 0.07 |  | 0.14 | | 0.22 | 0.06 | 0.08 | 0.41 | 0.02 |  | | 0.08 | 0.09 | | 0.02 | 0.03 | 0.81 | 0.06 |
| ***Deltaproteobacteria*** |  |  |  |  |  |  |  |  |  |  |  |  |  |  |  | |  |  |  |  |  |  | |  |  | |  |  |  |  |
| *BPC076* | 1.51 | 1.03 | 1.16 | 1.24 | 1.10 | 1.04 |  | 1.60 | 1.28 | 1.28 | 1.82 | 1.15 | 2.02 |  | 1.31 | | 1.40 | 1.13 | 1.48 | 0.55 | 1.04 |  | | 1.35 | 1.35 | | 0.70 | 1.43 | 1.39 | 0.73 |
| *Desulfarculales* | 1.29 | 0.65 | 0.94 | 1.01 | 1.21 | 0.55 |  | 0.93 | 0.59 | 1.04 | 1.05 | 1.78 | 0.95 |  | 0.93 | | 0.73 | 0.54 | 0.56 | 0.53 | 0.28 |  | | 0.90 | 0.73 | | 0.32 | 0.49 | 1.20 | 0.41 |
| *Desulfobacterales* | 1.74 | 0.78 | 0.90 | 0.79 | 1.86 | 0.83 |  | 1.35 | 2.36 | 0.97 | 1.15 | 2.41 | 0.84 |  | 1.22 | | 0.88 | 0.96 | 0.69 | 0.87 | 0.29 |  | | 1.15 | 0.96 | | 0.34 | 0.67 | 1.21 | 0.46 |
| *Desulfuromonadales* | 0.93 | 1.22 | 2.29 | 1.24 | 1.24 | 1.28 |  | 1.24 | 2.88 | 0.75 | 0.73 | 2.37 | 0.67 |  | 0.96 | | 0.55 | 1.68 | 0.71 | 0.48 | 0.17 |  | | 0.56 | 0.58 | | 0.15 | 0.15 | 0.70 | 0.35 |
| *Entotheonellales* | 1.22 | 0.95 | 1.37 | 0.27 | 2.29 | 0.82 |  | 2.76 | 2.15 | 1.12 | 1.23 | 0.90 | 0.57 |  | 3.73 | | 1.26 | 2.04 | 1.58 | 0.78 | 0.28 |  | | 1.70 | 1.73 | | 0.61 | 0.92 | 2.00 | 1.18 |
| *MBNT15* | 0.86 | 1.88 | 2.24 | 0.55 | 1.53 | 1.85 |  | 1.68 | 1.88 | 2.14 | 1.46 | 1.04 | 3.27 |  | 1.71 | | 1.26 | 2.88 | 2.57 | 0.58 | 0.78 |  | | 1.37 | 1.96 | | 1.10 | 1.39 | 1.52 | 2.32 |
| *Myxococcales* | 2.03 | 3.06 | 2.48 | 2.13 | 2.40 | 3.01 |  | 2.26 | 1.93 | 2.36 | 2.09 | 2.21 | 2.87 |  | 1.72 | | 2.29 | 2.21 | 2.01 | 0.94 | 0.73 |  | | 1.68 | 1.66 | | 0.53 | 0.85 | 1.33 | 1.23 |
| *NB1-j* | 0.27 | 0.46 | 0.52 | 0.23 | 0.52 | 0.57 |  | 0.23 | 0.58 | 0.46 | 0.34 | 0.30 | 1.14 |  | 0.39 | | 0.67 | 0.47 | 0.58 | 0.18 | 0.32 |  | | 0.29 | 0.52 | | 0.15 | 0.22 | 0.43 | 1.00 |
| *Syntrophobacterales* | 2.65 | 1.74 | 1.76 | 0.88 | 2.33 | 1.46 |  | 1.77 | 2.02 | 1.89 | 1.95 | 2.64 | 3.09 |  | 2.43 | | 2.99 | 2.30 | 2.34 | 1.12 | 1.14 |  | | 3.20 | 2.58 | | 1.16 | 1.91 | 2.74 | 2.38 |
| ***Epsilonproteobacteria*** |  |  |  |  |  |  |  |  |  |  |  |  |  |  |  | |  |  |  |  |  |  | |  |  | |  |  |  |  |
| *Campylobacterales* | 13.51 | 2.72 | 7.53 | 0.66 | 5.87 | 0.54 |  | 1.06 | 5.54 | 1.13 | 1.59 | 7.55 | 0.24 |  | 0.38 | | 0.39 | 0.04 | 0.02 | 2.57 | 0.05 |  | | 0.10 | 1.13 | | 0.05 | 0.31 | 0.59 | 0.24 |
| ***Gammaproteobacteria*** |  |  |  |  |  |  |  |  |  |  |  |  |  |  |  | |  |  |  |  |  |  | |  |  | |  |  |  |  |
| *Alteromonadales* | 0.93 | 1.18 | 1.15 | 0.86 | 1.03 | 1.29 |  | 0.81 | 1.38 | 1.19 | 1.26 | 0.87 | 1.36 |  | 0.81 | | 1.37 | 0.99 | 1.01 | 0.27 | 0.27 |  | | 1.08 | 1.02 | | 0.56 | 0.67 | 0.79 | 0.76 |
| *Methylococcales* | 0.68 | 0.15 | 0.37 | 0.22 | 1.49 | 0.31 |  | 0.55 | 0.18 | 0.44 | 0.59 | 1.59 | 0.64 |  | 0.89 | | 0.40 | 0.47 | 0.44 | 0.73 | 0.26 |  | | 0.58 | 0.38 | | 0.22 | 0.40 | 1.38 | 0.39 |
| *Oceanospirillales* | 0.28 | 0.08 | 0.05 | 0.13 | 0.35 | 0.11 |  | 0.44 | 0.52 | 0.07 | 0.24 | 0.19 | 0.50 |  | 0.64 | | 0.04 | 0.91 | 1.09 | 0.03 | 0.02 |  | | 0.73 | 0.55 | | 0.31 | 0.57 | 0.44 | 0.03 |
| *Pseudomonadales* | 0.06 | 2.28 | 6.54 | 3.06 | 0.15 | 0.12 |  | 0.23 | 0.72 | 0.38 | 0.52 | 0.02 | 0.46 |  | 0.05 | | 0.23 | 0.33 | 0.07 | 23.84 | 49.92 |  | | 9.00 | 7.70 | | 61.85 | 38.28 | 2.63 | 23.65 |
| *Xanthomonadales* | 1.61 | 1.77 | 2.57 | 1.60 | 2.12 | 2.60 |  | 1.25 | 2.87 | 3.46 | 2.11 | 1.51 | 3.70 |  | 3.38 | | 5.11 | 4.48 | 3.31 | 1.01 | 1.61 |  | | 3.67 | 3.66 | | 1.45 | 1.58 | 2.46 | 4.06 |
| ***Verrucomicrobia*** |  |  |  |  |  |  |  |  |  |  |  |  |  |  |  | |  |  |  |  |  |  | |  |  | |  |  |  |  |
| *S-BQ2-57* | 1.19 | 2.76 | 1.42 | 1.38 | 0.28 | 1.26 |  | 0.55 | 0.71 | 1.29 | 1.47 | 0.22 | 0.55 |  | 2.02 | | 1.05 | 1.04 | 1.18 | 0.15 | 0.21 |  | | 0.91 | 1.41 | | 0.21 | 0.33 | 0.20 | 0.62 |
| *Pedosphaerales* | 3.75 | 5.43 | 4.60 | 4.56 | 5.38 | 5.58 |  | 3.59 | 3.32 | 4.83 | 4.65 | 5.40 | 4.82 |  | 3.54 | | 5.39 | 4.69 | 4.92 | 1.78 | 2.08 |  | | 3.36 | 3.81 | | 1.62 | 2.12 | 3.54 | 4.13 |
| *Chthoniobacterales* | 0.42 | 0.39 | 0.49 | 0.28 | 0.47 | 0.67 |  | 0.27 | 2.26 | 0.30 | 0.63 | 0.17 | 0.68 |  | 1.66 | | 1.14 | 0.70 | 1.04 | 0.22 | 0.48 |  | | 0.88 | 1.82 | | 0.29 | 0.56 | 0.60 | 1.89 |
| ***WS3*** |  |  |  |  |  |  |  |  |  |  |  |  |  |  |  | |  |  |  |  |  |  | |  |  | |  |  |  |  |
| *Sediment-1* | 0.77 | 0.89 | 0.95 | 0.61 | 0.76 | 1.60 |  | 0.75 | 0.89 | 1.03 | 1.03 | 0.53 | 1.27 |  | 1.10 | | 1.43 | 0.80 | 1.24 | 0.28 | 0.72 |  | | 0.95 | 1.03 | | 0.31 | 0.67 | 0.83 | 1.26 |

**Table S6.** Abundances of bacterial family (%) detected by Illumina sequencing in each sediment samples (largest relative abundance more than 1%)

| Taxon | Spring | | | | | | | Summer | | | | | | | Fall | | | | | |  | Winter | | | | | |
| --- | --- | --- | --- | --- | --- | --- | --- | --- | --- | --- | --- | --- | --- | --- | --- | --- | --- | --- | --- | --- | --- | --- | --- | --- | --- | --- | --- |
|  | 5# | 8# | 11# | 15# | 18# | 19# |  | 5# | 8# | 11# | 15# | 18# | 19# |  | 5# | 8# | 11# | 15# | 18# | 19# |  | 5# | 8# | 11# | 15# | 18# | 19# |
| ***Actinobacteria*** |  |  |  |  |  |  |  |  |  |  |  |  |  |  |  |  |  |  |  |  |  |  |  |  |  |  |  |
| *ACK-M1* | 0.08 | 0.20 | 0.09 | 0.51 | 0.05 | 1.12 |  | 0.07 | 0.04 | 0.16 | 0.12 | 0.03 | 0.07 |  | 0.02 | 0.06 | 0.03 | 0.03 | 0.01 | — |  | 0.04 | 0.03 | 0.02 | 0.02 | 0.01 | 0.04 |
| ***Bacteroidetes*** |  |  |  |  |  |  |  |  |  |  |  |  |  |  |  |  |  |  |  |  |  |  |  |  |  |  |  |
| *Bacteroidaceae* | — | 0.07 | — | 0.05 | 0.01 | — |  | 0.01 | 1.51 | 0.01 | 0.01 | 0.02 | — |  | — | — | 0.01 | — | 0.01 | 0.18 |  | — | 0.03 | — | 0.01 | 0.02 | 0.01 |
| *Cytophagaceae* | 0.82 | 3.03 | 1.75 | 5.15 | 2.13 | 4.85 |  | 0.80 | 0.96 | 2.23 | 1.89 | 2.19 | 1.90 |  | 0.42 | 0.92 | 1.04 | 0.87 | 0.63 | 0.26 |  | 0.51 | 0.49 | 0.29 | 0.35 | 1.35 | 0.71 |
| *Cryomorphaceae* | 0.08 | 0.15 | 0.19 | 1.23 | 0.15 | 0.36 |  | 0.16 | 0.09 | 0.17 | 0.27 | 0.10 | 0.16 |  | 0.08 | 0.06 | 0.05 | 0.08 | 0.05 | 0.02 |  | 0.06 | 0.03 | 0.02 | 0.05 | 0.13 | 0.02 |
| *Flavobacteriaceae* | 0.45 | 0.98 | 0.43 | 2.21 | 0.68 | 1.41 |  | 1.20 | 0.91 | 0.38 | 0.68 | 0.67 | 0.42 |  | 0.31 | 0.75 | 0.17 | 0.21 | 23.39 | 9.43 |  | 0.26 | 0.29 | 0.14 | 0.15 | 0.52 | 0.21 |
| *Chitinophagaceae* | 1.11 | 0.94 | 1.59 | 1.90 | 2.08 | 2.39 |  | 0.84 | 1.39 | 1.99 | 2.08 | 1.44 | 3.01 |  | 1.91 | 2.51 | 2.76 | 3.18 | 1.04 | 1.14 |  | 2.01 | 1.70 | 1.06 | 1.62 | 2.24 | 2.16 |
| *Saprospiraceae* | 0.67 | 1.18 | 1.41 | 2.54 | 1.48 | 1.83 |  | 0.72 | 1.19 | 1.88 | 1.72 | 0.73 | 1.88 |  | 0.81 | 1.86 | 2.77 | 2.31 | 0.56 | 1.06 |  | 1.08 | 1.25 | 0.99 | 1.24 | 1.45 | 1.49 |
| ***Chlorobi*** |  |  |  |  |  |  |  |  |  |  |  |  |  |  |  |  |  |  |  |  |  |  |  |  |  |  |  |
| *Ignavibacteriaceae* | 0.90 | 0.43 | 0.47 | 0.70 | 0.65 | 0.42 |  | 1.01 | 0.52 | 0.76 | 0.94 | 0.96 | 0.60 |  | 1.02 | 0.77 | 0.56 | 0.63 | 0.36 | 0.30 |  | 0.92 | 0.81 | 0.41 | 0.60 | 0.48 | 0.36 |
| ***Cyanobacteria*** |  |  |  |  |  |  |  |  |  |  |  |  |  |  |  |  |  |  |  |  |  |  |  |  |  |  |  |
| *Synechococcaceae* | 0.14 | 0.06 | 0.30 | 0.06 | 1.46 | 0.75 |  | 0.09 | 0.33 | 0.19 | 0.17 | 0.24 | 0.51 |  | 0.45 | 0.46 | 2.23 | 1.66 | 0.31 | 0.41 |  | 0.39 | 0.60 | 0.43 | 0.61 | 2.02 | 2.67 |
| ***Firmicutes*** |  |  |  |  |  |  |  |  |  |  |  |  |  |  |  |  |  |  |  |  |  |  |  |  |  |  |  |
| *Clostridiaceae* | 0.24 | 0.32 | 0.27 | 0.07 | 0.38 | 0.04 |  | 0.40 | 1.43 | 0.72 | 0.54 | 0.93 | 0.39 |  | 0.68 | 0.83 | 1.80 | 1.52 | 1.09 | 1.00 |  | 3.42 | 3.34 | 1.30 | 2.19 | 11.23 | 0.97 |
| *Peptostreptococcaceae* | 0.19 | 0.13 | 0.09 | 0.01 | 0.08 | 0.02 |  | 0.48 | 0.22 | 0.23 | 0.79 | 0.21 | 0.22 |  | 0.35 | 1.01 | 1.76 | 3.72 | 0.07 | 0.39 |  | 0.31 | 0.84 | 0.26 | 1.20 | 1.63 | 0.93 |
| ***Nitrospirae*** |  |  |  |  |  |  |  |  |  |  |  |  |  |  |  |  |  |  |  |  |  |  |  |  |  |  |  |
| *FW* | 1.23 | 1.94 | 0.96 | 1.62 | 0.60 | 1.01 |  | 6.10 | 1.15 | 1.30 | 1.22 | 1.64 | 0.56 |  | 2.63 | 0.46 | 1.04 | 0.68 | 0.37 | 0.11 |  | 0.54 | 0.62 | 0.10 | 0.32 | 0.37 | 1.23 |
| *Nitrospiraceae* | 0.32 | 1.42 | 0.86 | 2.22 | 0.21 | 1.68 |  | 0.28 | 0.38 | 0.80 | 0.77 | 0.23 | 1.11 |  | 0.15 | 0.69 | 0.33 | 0.37 | 0.07 | 0.10 |  | 0.09 | 0.21 | 0.07 | 0.16 | 0.12 | 0.32 |
| *Thermodesulfovibrionaceae* | 8.25 | 5.82 | 4.67 | 6.49 | 7.09 | 3.99 |  | 11.82 | 7.16 | 6.14 | 7.10 | 6.43 | 8.39 |  | 9.66 | 4.83 | 3.84 | 5.10 | 2.40 | 2.70 |  | 8.84 | 8.14 | 2.44 | 5.72 | 8.86 | 8.25 |
| ***Planctomycetes*** |  |  |  |  |  |  |  |  |  |  |  |  |  |  |  |  |  |  |  |  |  |  |  |  |  |  |  |
| *Pirellulaceae* | 0.73 | 0.65 | 0.52 | 0.44 | 0.46 | 0.53 |  | 0.56 | 0.96 | 0.40 | 0.58 | 0.22 | 0.37 |  | 1.13 | 1.09 | 1.06 | 1.25 | 0.20 | 0.32 |  | 1.09 | 1.23 | 0.65 | 0.89 | 0.71 | 0.92 |
| ***Betaproteobacteria*** |  |  |  |  |  |  |  |  |  |  |  |  |  |  |  |  |  |  |  |  |  |  |  |  |  |  |  |
| *Comamonadaceae* | 0.43 | 1.49 | 1.23 | 1.50 | 1.30 | 2.12 |  | 0.76 | 0.90 | 1.06 | 0.92 | 0.82 | 1.08 |  | 0.53 | 0.72 | 0.90 | 0.64 | 1.95 | 0.61 |  | 0.57 | 0.42 | 0.36 | 0.42 | 0.97 | 0.63 |
| *Oxalobacteraceae* | 0.05 | 0.09 | 0.06 | 0.30 | 0.09 | 0.15 |  | 0.04 | 0.03 | 0.01 | 0.04 | 0.02 | 0.03 |  | 0.02 | 0.03 | 0.01 | 0.02 | 7.03 | 0.87 |  | 0.03 | 0.02 | 0.03 | 0.01 | 0.01 | — |
| *Gallionellaceae* | 1.95 | 1.89 | 1.00 | 1.22 | 1.20 | 0.69 |  | 1.82 | 1.12 | 2.85 | 2.45 | 1.36 | 0.51 |  | 0.63 | 0.59 | 0.20 | 0.33 | 0.24 | 0.03 |  | 0.36 | 0.41 | 0.11 | 0.38 | 0.21 | 0.11 |
| *Hydrogenophilaceae* | 0.92 | 1.22 | 0.37 | 0.43 | 1.71 | 0.47 |  | 1.49 | 0.75 | 0.74 | 0.86 | 2.45 | 0.42 |  | 0.86 | 0.85 | 0.20 | 0.30 | 4.56 | 1.02 |  | 0.27 | 0.59 | 0.05 | 0.19 | 0.80 | 0.28 |
| *Rhodocyclaceae* | 1.09 | 1.22 | 0.81 | 0.67 | 0.81 | 0.68 |  | 0.93 | 1.41 | 0.57 | 0.91 | 0.96 | 0.23 |  | 0.45 | 0.41 | 0.36 | 0.24 | 0.25 | 0.05 |  | 0.27 | 0.24 | 0.29 | 0.14 | 0.32 | 0.16 |
| ***Deltaproteobacteria*** |  |  |  |  |  |  |  |  |  |  |  |  |  |  |  |  |  |  |  |  |  |  |  |  |  |  |  |
| *Desulfarculaceae* | 1.29 | 0.65 | 0.94 | 1.01 | 1.21 | 0.55 |  | 0.93 | 0.59 | 1.04 | 1.05 | 1.78 | 0.95 |  | 0.93 | 0.73 | 0.54 | 0.56 | 0.53 | 0.28 |  | 0.90 | 0.73 | 0.32 | 0.49 | 1.20 | 0.41 |
| *Desulfobacteraceae* | 1.24 | 0.55 | 0.57 | 0.63 | 1.22 | 0.51 |  | 0.80 | 0.76 | 0.78 | 0.87 | 1.70 | 0.73 |  | 0.81 | 0.69 | 0.57 | 0.58 | 0.65 | 0.24 |  | 0.87 | 0.77 | 0.30 | 0.60 | 0.92 | 0.35 |
| *Desulfobulbaceae* | 0.49 | 0.22 | 0.31 | 0.15 | 0.54 | 0.32 |  | 0.53 | 1.59 | 0.19 | 0.25 | 0.69 | 0.10 |  | 0.40 | 0.19 | 0.38 | 0.11 | 0.20 | 0.05 |  | 0.28 | 0.19 | 0.04 | 0.07 | 0.27 | 0.11 |
| *Desulfuromonadaceae* | 0.26 | 0.55 | 0.69 | 0.49 | 0.42 | 0.27 |  | 0.26 | 1.05 | 0.24 | 0.26 | 0.48 | 0.26 |  | 0.32 | 0.23 | 0.36 | 0.30 | 0.14 | 0.06 |  | 0.13 | 0.23 | 0.05 | 0.05 | 0.18 | 0.15 |
| *Geobacteraceae* | 0.38 | 0.38 | 1.25 | 0.53 | 0.62 | 0.90 |  | 0.76 | 1.02 | 0.27 | 0.32 | 1.63 | 0.29 |  | 0.35 | 0.17 | 0.71 | 0.21 | 0.27 | 0.08 |  | 0.16 | 0.17 | 0.06 | 0.07 | 0.35 | 0.12 |
| *Syntrophaceae* | 1.54 | 0.76 | 0.59 | 0.25 | 1.06 | 0.40 |  | 0.78 | 0.71 | 0.56 | 0.85 | 1.34 | 0.87 |  | 1.13 | 0.85 | 0.78 | 0.80 | 0.52 | 0.31 |  | 1.76 | 1.06 | 0.46 | 0.86 | 1.18 | 0.46 |
| *Syntrophobacteraceae* | 1.04 | 0.95 | 1.12 | 0.59 | 1.10 | 0.99 |  | 0.95 | 1.28 | 1.26 | 1.05 | 0.93 | 2.12 |  | 1.24 | 2.12 | 1.45 | 1.50 | 0.53 | 0.78 |  | 1.35 | 1.47 | 0.68 | 1.01 | 1.34 | 1.86 |
| *Campylobacteraceae* | — | 0.01 | 7.08 | — | — | 0.02 |  | 0.01 | 4.46 | — | 0.24 | 0.01 | 0.01 |  | 0.11 | 0.01 | — | — | 0.01 | — |  | — | — | — | — | — | — |
| ***Epsilonproteobacteria*** |  |  |  |  |  |  |  |  |  |  |  |  |  |  |  |  |  |  |  |  |  |  |  |  |  |  |  |
| *Helicobacteraceae* | 13.51 | 2.71 | 0.46 | 0.66 | 5.87 | 0.52 |  | 1.05 | 1.08 | 1.13 | 1.35 | 7.53 | 0.23 |  | 0.27 | 0.38 | 0.04 | 0.01 | 2.55 | 0.05 |  | 0.10 | 1.12 | 0.05 | 0.31 | 0.59 | 0.24 |
| ***Gammaproteobacteria*** |  |  |  |  |  |  |  |  |  |  |  |  |  |  |  |  |  |  |  |  |  |  |  |  |  |  |  |
| *Halomonadaceae* | 0.28 | 0.08 | 0.05 | 0.13 | 0.35 | 0.11 |  | 0.44 | 0.52 | 0.07 | 0.24 | 0.19 | 0.50 |  | 0.64 | 0.04 | 0.91 | 1.09 | 0.03 | 0.02 |  | 0.73 | 0.55 | 0.31 | 0.57 | 0.44 | 0.03 |
| *Moraxellaceae* | 0.05 | 1.64 | 5.75 | 2.04 | 0.12 | 0.07 |  | 0.19 | 0.34 | 0.35 | 0.35 | 0.02 | 0.44 |  | 0.03 | 0.06 | 0.04 | 0.07 | 17.49 | 48.31 |  | 8.98 | 7.67 | 61.78 | 38.18 | 2.63 | 23.64 |
| *Pseudomonadaceae* | 0.01 | 0.64 | 0.78 | 1.03 | 0.03 | 0.04 |  | 0.04 | 0.39 | 0.03 | 0.16 | 0.01 | 0.02 |  | 0.02 | 0.16 | 0.29 | 0.01 | 6.35 | 1.61 |  | 0.02 | 0.03 | 0.07 | 0.10 | — | — |
| *Sinobacteraceae* | 1.44 | 1.16 | 2.02 | 0.75 | 1.68 | 1.79 |  | 0.99 | 2.12 | 2.71 | 1.52 | 1.28 | 2.67 |  | 3.08 | 3.82 | 4.11 | 2.75 | 0.74 | 1.15 |  | 3.37 | 3.31 | 1.29 | 1.36 | 2.12 | 3.20 |
| *Xanthomonadaceae* | 0.15 | 0.54 | 0.43 | 0.79 | 0.40 | 0.66 |  | 0.26 | 0.70 | 0.56 | 0.49 | 0.21 | 0.59 |  | 0.28 | 1.04 | 0.26 | 0.33 | 0.27 | 0.28 |  | 0.28 | 0.24 | 0.12 | 0.14 | 0.30 | 0.25 |
| ***Verrucomicrobia*** |  |  |  |  |  |  |  |  |  |  |  |  |  |  |  |  |  |  |  |  |  |  |  |  |  |  |  |
| *Ellin515* | 1.62 | 1.43 | 1.32 | 0.78 | 2.29 | 1.15 |  | 1.52 | 1.41 | 1.34 | 1.48 | 2.52 | 1.97 |  | 1.70 | 1.92 | 1.88 | 1.90 | 0.69 | 1.03 |  | 1.61 | 1.75 | 0.74 | 0.97 | 1.65 | 1.77 |
| *auto67_4W* | 0.67 | 1.78 | 1.10 | 1.55 | 1.16 | 1.69 |  | 0.77 | 0.65 | 1.08 | 1.20 | 1.03 | 0.85 |  | 0.46 | 1.23 | 0.69 | 0.87 | 0.41 | 0.36 |  | 0.46 | 0.71 | 0.21 | 0.38 | 0.59 | 0.56 |
| *Chthoniobacteraceae* | 0.42 | 0.39 | 0.49 | 0.28 | 0.47 | 0.67 |  | 0.27 | 2.26 | 0.30 | 0.63 | 0.17 | 0.68 |  | 1.66 | 1.13 | 0.70 | 1.04 | 0.21 | 0.48 |  | 0.88 | 1.82 | 0.29 | 0.56 | 0.60 | 1.89 |
| ***WS3*** |  |  |  |  |  |  |  |  |  |  |  |  |  |  |  |  |  |  |  |  |  |  |  |  |  |  |  |
| *PRR-10* | 0.46 | 0.58 | 0.57 | 0.39 | 0.41 | 1.15 |  | 0.38 | 0.50 | 0.54 | 0.64 | 0.23 | 0.73 |  | 0.61 | 0.88 | 0.46 | 0.73 | 0.14 | 0.49 |  | 0.49 | 0.62 | 0.18 | 0.39 | 0.47 | 0.77 |

—: not detected

**Table S7.** Abundances of bacterial genera (%) detected by Illumina sequencing in each sediment samples (largest relative abundance more than 1%)

| Taxon | Spring | | | | | |  | Summer | | | | | |  | Fall | | | | | |  | Winter | | | | | |
| --- | --- | --- | --- | --- | --- | --- | --- | --- | --- | --- | --- | --- | --- | --- | --- | --- | --- | --- | --- | --- | --- | --- | --- | --- | --- | --- | --- |
|  | 5# | 8# | 11# | 15# | 18# | 19# |  | 5# | 8# | 11# | 15# | 18# | 19# |  | 5# | 8# | 11# | 15# | 18# | 19# |  | 5# | 8# | 11# | 15# | 18# | 19# |
| ***Bacteroidetes*** |  |  |  |  |  |  |  |  |  |  |  |  |  |  |  |  |  |  |  |  |  |  |  |  |  |  |  |
| *Bacteroides* | — | 0.07 | — | 0.05 | 0.01 | — |  | 0.01 | 1.51 | 0.01 | 0.01 | 0.02 | — |  | — | — | 0.01 | — | 0.01 | 0.18 |  | — | 0.03 | — | 0.01 | 0.02 | 0.01 |
| *Flavobacterium* | 0.37 | 0.92 | 0.34 | 1.98 | 0.48 | 1.27 |  | 1.10 | 0.76 | 0.30 | 0.55 | 0.34 | 0.25 |  | 0.23 | 0.55 | 0.13 | 0.13 | 23.2 | 9.35 |  | 0.21 | 0.21 | 0.11 | 0.11 | 0.34 | 0.14 |
| ***Cyanobacteria*** |  |  |  |  |  |  |  |  |  |  |  |  |  |  |  |  |  |  |  |  |  |  |  |  |  |  |  |
| *Synechococcus* | 0.13 | 0.05 | 0.30 | 0.06 | 1.37 | 0.62 |  | 0.08 | 0.29 | 0.19 | 0.16 | 0.21 | 0.47 |  | 0.42 | 0.27 | 2.17 | 1.57 | 0.27 | 0.31 |  | 0.37 | 0.50 | 0.39 | 0.56 | 1.69 | 2.33 |
| ***Firmicutes*** |  |  |  |  |  |  |  |  |  |  |  |  |  |  |  |  |  |  |  |  |  |  |  |  |  |  |  |
| *Clostridium* | 0.30 | 0.32 | 0.27 | 0.06 | 0.30 | 0.04 |  | 0.49 | 1.47 | 0.77 | 0.71 | 0.77 | 0.38 |  | 0.77 | 1.34 | 2.46 | 4.57 | 1.10 | 1.14 |  | 3.40 | 3.40 | 1.29 | 2.28 | 9.24 | 1.00 |
| *Tepidibacter* | 0.08 | 0.05 | 0.04 | — | 0.02 | 0.01 |  | 0.17 | 0.04 | 0.10 | 0.42 | 0.07 | 0.12 |  | 0.16 | 0.36 | 0.86 | 0.57 | 0.04 | 0.18 |  | 0.23 | 0.73 | 0.18 | 1.03 | 0.94 | 0.85 |
| ***Nitrospirae*** |  |  |  |  |  |  |  |  |  |  |  |  |  |  |  |  |  |  |  |  |  |  |  |  |  |  |  |
| *4-29* | 1.23 | 1.94 | 0.96 | 1.62 | 0.60 | 1.01 |  | 6.10 | 1.15 | 1.30 | 1.22 | 1.64 | 0.56 |  | 2.63 | 0.46 | 1.04 | 0.68 | 0.37 | 0.11 |  | 0.54 | 0.62 | 0.10 | 0.32 | 0.37 | 0.15 |
| *Nitrospira* | 0.32 | 1.40 | 0.84 | 2.21 | 0.20 | 1.57 |  | 0.28 | 0.37 | 0.79 | 0.75 | 0.22 | 1.03 |  | 0.15 | 0.69 | 0.33 | 0.36 | 0.07 | 0.10 |  | 0.09 | 0.20 | 0.06 | 0.16 | 0.12 | 0.21 |
| *GOUTA19* | 2.73 | 3.76 | 2.31 | 3.75 | 1.56 | 2.13 |  | 5.95 | 3.81 | 3.20 | 3.49 | 1.16 | 2.51 |  | 3.86 | 1.97 | 1.84 | 2.27 | 0.54 | 0.73 |  | 2.56 | 2.99 | 0.99 | 1.71 | 1.52 | 1.15 |
| *HB118* | 0.43 | 0.10 | 0.28 | 0.17 | 0.43 | 0.03 |  | 0.69 | 0.32 | 0.35 | 0.30 | 0.84 | 0.37 |  | 0.98 | 0.16 | 0.67 | 0.35 | 0.07 | 0.08 |  | 0.78 | 0.73 | 0.19 | 0.25 | 1.04 | 0.18 |
| *LCP-6* | 4.21 | 1.04 | 1.23 | 1.60 | 4.73 | 0.93 |  | 3.78 | 2.11 | 1.60 | 2.25 | 4.19 | 4.04 |  | 3.84 | 1.76 | 0.82 | 1.58 | 1.66 | 1.39 |  | 4.66 | 3.52 | 0.89 | 2.97 | 5.79 | 1.15 |
| ***Betaproteobacteria*** |  |  |  |  |  |  |  |  |  |  |  |  |  |  |  |  |  |  |  |  |  |  |  |  |  |  |  |
| *Janthinobacterium* | — | 0.03 | 0.01 | 0.09 | — | 0.02 |  | — | — | — | — | — | — |  | — | 0.01 | — | — | 5.81 | 0.65 |  | — | — | — | — | — | — |
| *Gallionella* | 1.95 | 1.89 | 1.00 | 1.22 | 1.20 | 0.69 |  | 1.82 | 1.12 | 2.85 | 2.45 | 1.36 | 0.51 |  | 0.63 | 0.59 | 0.20 | 0.33 | 0.24 | 0.03 |  | 0.36 | 0.41 | 0.11 | 0.38 | 0.21 | 0.11 |
| *Thiobacillus* | 0.92 | 1.22 | 0.37 | 0.43 | 1.70 | 0.47 |  | 1.49 | 0.75 | 0.74 | 0.86 | 2.45 | 0.41 |  | 0.86 | 0.85 | 0.20 | 0.30 | 4.56 | 1.02 |  | 0.27 | 0.59 | 0.05 | 0.19 | 0.80 | 0.28 |
| ***Deltaproteobacteria*** |  |  |  |  |  |  |  |  |  |  |  |  |  |  |  |  |  |  |  |  |  |  |  |  |  |  |  |
| *Desulfobulbus* | 0.16 | 0.08 | 0.19 | 0.03 | 0.16 | 0.04 |  | 0.07 | 1.40 | 0.08 | 0.12 | 0.28 | 0.04 |  | 0.08 | 0.06 | 0.18 | 0.04 | 0.06 | 0.02 |  | 0.04 | 0.03 | 0.01 | 0.01 | 0.06 | 0.01 |
| *Geobacter* | 0.28 | 0.31 | 0.87 | 0.32 | 0.53 | 0.62 |  | 0.64 | 0.75 | 0.16 | 0.25 | 1.53 | 0.23 |  | 0.30 | 0.12 | 0.63 | 0.14 | 0.26 | 0.05 |  | 0.13 | 0.11 | 0.04 | 0.04 | 0.32 | 0.07 |
| *Desulfobacca* | 1.22 | 0.68 | 0.46 | 0.13 | 0.60 | 0.27 |  | 0.58 | 0.59 | 0.42 | 0.67 | 0.47 | 0.71 |  | 0.71 | 0.65 | 0.53 | 0.58 | 0.29 | 0.24 |  | 1.47 | 0.81 | 0.40 | 0.74 | 0.89 | 0.37 |
| ***Epsilonproteobacteria*** |  |  |  |  |  |  |  |  |  |  |  |  |  |  |  |  |  |  |  |  |  |  |  |  |  |  |  |
| *Arcobacter* | — | 0.01 | 7.08 | — | — | 0.01 |  | — | 4.46 | — | 0.24 | — | 0.01 |  | 0.11 | 0.01 | — | — | 0.01 | — |  | — | — | — | — | — | — |
| *Sulfuricurvum* | 12.67 | 2.60 | 0.45 | 0.66 | 5.72 | 0.42 |  | 0.87 | 1.04 | 1.12 | 1.34 | 6.87 | 0.22 |  | 0.24 | 0.36 | 0.03 | 0.01 | 2.23 | 0.04 |  | 0.08 | 1.08 | 0.04 | 0.30 | 0.54 | 0.19 |
| ***Gammaproteobacteria*** |  |  |  |  |  |  |  |  |  |  |  |  |  |  |  |  |  |  |  |  |  |  |  |  |  |  |  |
| *Halomonas* | 0.27 | 0.04 | 0.03 | 0.09 | 0.32 | 0.05 |  | 0.43 | 0.50 | 0.05 | 0.21 | 0.14 | 0.48 |  | 0.63 | 0.03 | 0.91 | 1.08 | 0.02 | 0.02 |  | 0.73 | 0.55 | 0.31 | 0.57 | 0.42 | 0.02 |
| *Acinetobacter* | 0.02 | 0.98 | 5.64 | 0.06 | 0.11 | 0.01 |  | 0.06 | 0.23 | 0.19 | 0.07 | 0.01 | 0.42 |  | 0.02 | 0.03 | 0.03 | 0.06 | 17.4 | 48.21 |  | 8.98 | 7.67 | 61.77 | 38.16 | 2.63 | 23.64 |
| *Pseudomonas* | 0.01 | 0.58 | 0.57 | 0.19 | 0.01 | 0.01 |  | 0.01 | 0.29 | 0.02 | 0.15 | 0.01 | 0.01 |  | 0.02 | 0.16 | 0.29 | 0.01 | 6.35 | 1.61 |  | 0.02 | — | 0.04 | 0.05 | — | — |
| ***Verrucomicrobia*** |  |  |  |  |  |  |  |  |  |  |  |  |  |  |  |  |  |  |  |  |  |  |  |  |  |  |  |
| *Xiphinematobacter* | 0.30 | 0.22 | 0.20 | 0.14 | 0.13 | 0.25 |  | 0.17 | 1.99 | 0.16 | 0.45 | 0.04 | 0.29 |  | 1.55 | 0.73 | 0.49 | 0.70 | 0.03 | 0.15 |  | 0.66 | 1.46 | 0.12 | 0.29 | 0.16 | 1.24 |

—: not detected


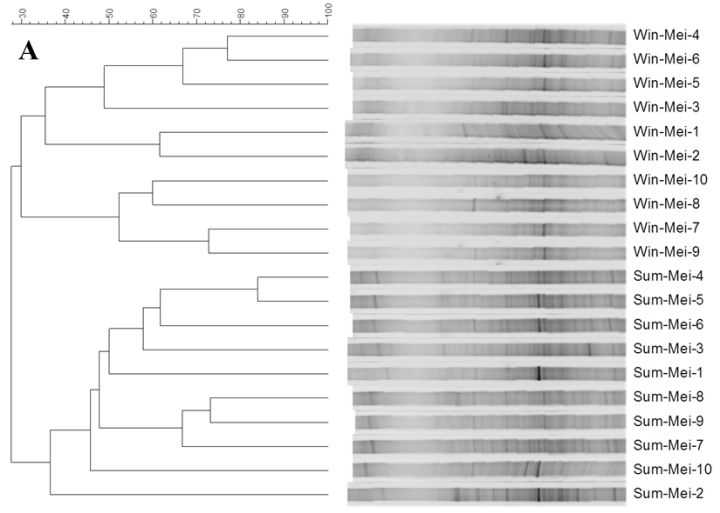


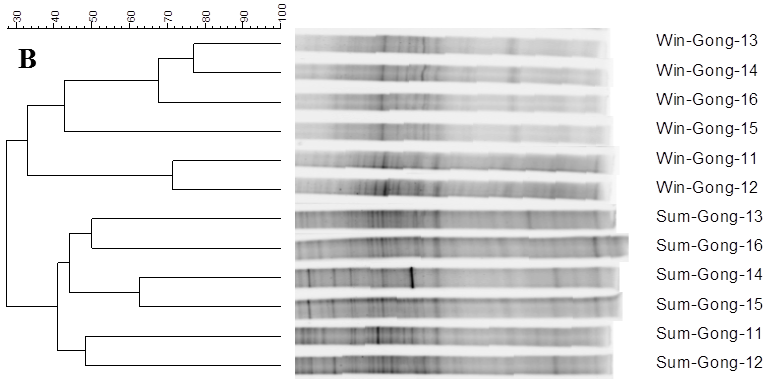


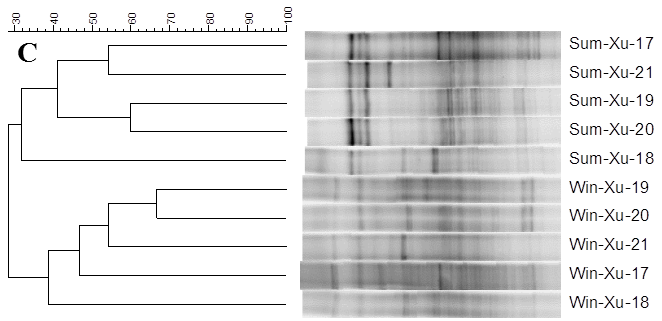


**Fig. S1.** Dendrograms obtained by UPGMA clustering of DGGE banding patterns from Meiliang Bay (A), Gonghu Bay (B) and Xukou Bay (C). The samples were marked as ‘Season-Lake Region-Site number’, for example, Spr-Mei-5 was the sample collected from site 5 in the sediment of Meiliang Bay in spring.

1. * Corresponding author: Xiaohong Ruan. Nanjing University, 163 Xianlin Rd, Qixia District, Nanjing 210093, Jiangsu Province, P. R. China. Email: ruanxh@nju.edu.cn. Phone: +86-025-83597099. Fax: +86-025-83597099 [↑](#footnote-ref-1)
